# A Prediction-Enhanced Polynomial Kalman Filter Using Higher-Order Predictive Representations for State Estimation

**DOI:** 10.3390/s26185871

**Published:** 2026-09-16

**Authors:** Jing Long, Haiyang Zhang, Chenglin Wen

**Affiliations:** 1 College of Automation, Guangdong University of Petrochemical Technology, Maoming 525000, China; long@gdupt.edu.cn; 2 School of Automation and Intelligence, Beijing Jiaotong University, Beijing 100044, China; 25110021@bjtu.edu.cn; 3 Guangdong Provincial Key Laboratory of Petrochemical Equipment Fault Diagnosis, Guangdong University of Petrochemical Technology, Maoming 525000, China

**Keywords:** prediction-enhanced polynomial Kalman filter, higher-order polynomial predictive representation, Kronecker augmentation, nonlinear predictive transformation, weighted least squares

## Abstract

The conventional Kalman filter operates primarily in the original first-order state-prediction space, which limits the explicit utilization of higher-order state-coupling and predictive statistical structures. To enlarge the candidate prediction space, this study proposes a Prediction-Enhanced Polynomial Kalman Filter (PEPKF). Through Kronecker augmentation, the original one-step prediction is extended to second-, third-, and higher-order polynomial representation spaces, introducing nonlinear internal predictive transformations while preserving the linear physical state-space model. The resulting higher-order quantities are formulated as equivalent predictive pseudo-observations and fused with the original prediction through weighted least squares to construct an enhanced prior, followed by the standard Kalman measurement update. Numerical simulation results show that PEPKF achieves better state-estimation performance than the conventional KF, thereby demonstrating the effectiveness of the proposed method.

## 1. Introduction

State estimation is a fundamental technique in intelligent sensing, autonomous driving [1,2], parameter estimation [3,4], system identification [5,6], robotics, and aerospace engineering [7]. Its objective is to infer internal system states from noisy and uncertain sensor observations. In practical sensing systems, measurement noise, limited sensor accuracy, environmental disturbances, and model uncertainty may degrade estimation reliability. Therefore, effective use of limited sensor information remains an important issue in sensor information fusion and dynamic process monitoring [8,9,10,11].

Recent studies further demonstrate the broad applicability of recursive state-estimation and filtering methods in sensor-driven systems. Liang et al. investigated covariance-optimized particle filtering for cooperative localization of autonomous underwater vehicles; Zhai et al. employed Kalman filtering for online Jacobian-error estimation and compensation in continuum manipulators; and Zhang et al. applied continuous Kalman estimation to finger-kinematics tracking from surface electromyography signals [12,13,14]. These studies illustrate the continuing importance of state estimation in localization, robotic control, and sensor-based motion estimation.

For accurately specified linear systems with Gaussian process and measurement noise, the Kalman filter provides the conditional-mean MMSE estimate based on the original measurement information. In this study, the physical system remains linear; however, the internal predictive representation is extended beyond the conventional first-order state representation by nonlinear polynomial transformations, including squared-state, cross-product, and higher-order Kronecker mappings [15,16]. In practical applications, however, model uncertainty, imperfect noise characterization, and more complex prediction-error statistics may affect the predictive description. Adaptive and modified Kalman filtering methods have therefore been developed to improve estimation performance under non-ideal statistical conditions [17,18,19].

Another direction is to introduce higher-order state representations into the estimation process. Polynomial filtering incorporates higher-order combinations of state variables through polynomial approximation and state augmentation [20,21,22], while higher-order sigma-point methods improve the approximation of transformed random-variable statistics by exploiting higher-order moment information [23]. High-order EKF/KF methods based on nonlinear expansions and Kronecker-product transformations have also been investigated [24,25]. The Kronecker product provides a systematic mathematical representation of multi-order coupling relationships among state variables [26,27].

Most existing high-order filtering approaches either consider nonlinear physical state or measurement models or perform the filtering recursion directly in an augmented high-dimensional state space. In contrast, relatively limited attention has been paid to enlarging only the predictive representation space while retaining the original physical model and measurement-update structure.

To address this issue, this paper proposes a prediction-enhanced polynomial Kalman filter (PEPKF). The physical state-space model remains linear, while the conventional first-order prediction is extended to second-, third-, and higher-order polynomial representations through Kronecker augmentation. These nonlinear internal predictive transformations generate equivalent higher-order predictive pseudo-observations. Their higher-order prediction-error statistics and cross-order correlations are approximated using sigma-point sampling.

Importantly, the Kronecker augmentation is not a translation, rotation, or rearrangement of the first-order prediction within the same state space. Rather, it maps the original $n_{x}$-dimensional prediction into higher-order characteristic spaces. For example, the second-order representation belongs to an $n_{x}^{2}$-dimensional space in which squared-state terms and cross-state coupling characteristics are explicitly represented. The generated higher-order equations may still contain components already represented by lower-order predictions; these components are subsequently removed through Gram–Schmidt orthogonalization, and the retained effective components are used in predictive fusion.

Since the original prediction and the higher-order predictive representations originate from the same underlying prediction process, their errors are generally correlated. Sequential Gram–Schmidt orthogonalization is therefore introduced to remove correlated and redundant components before weighted least-squares fusion. The remaining non-redundant predictive representations are fused to construct an enhanced prior, after which the standard Kalman measurement-update structure is retained. Thus, PEPKF provides a prediction-enhancement framework that exploits higher-order predictive structures without altering the underlying linear physical system or sensor measurement model.

The main contributions of this paper are summarized as follows:

1. A nonlinear higher-order predictive-representation framework is developed that enlarges the internal prediction space while retaining the original linear physical model and the standard Kalman measurement-update structure.
2. Kronecker-product-based polynomial representations are constructed to describe second-, third-, and higher-order predictive structures, with sigma-point sampling used to approximate the associated higher-order statistics and cross-order correlations.
3. A correlation-aware multi-order predictive-fusion mechanism is developed. Sequential Gram–Schmidt orthogonalization removes correlated and redundant components, after which the retained predictive representations are fused by weighted least squares to construct the enhanced prior.

## 2. Linear System Description

Linear systems constitute one of the most fundamental and important classes of systems in control theory and state-estimation research. Their dynamic behavior can be described by linear combinations of state variables and input variables. Consider the following discrete-time linear system:

(1) $$\left\{\begin{array}{cc} x(k+1) & =A(k+1,k)x(k)+w(k),\\ y(k+1) & =H(k+1)x(k+1)+v(k+1), \end{array}\right.$$

where $x\left(k\right)\in R^{n_{x}}$ denotes the system state vector, $A\left(k+1,k\right)\in R^{n_{x}\times n_{x}}$ is the state-transition matrix, $y\left(k+1\right)\in R^{n_{y}}$ denotes the measurement vector of the state x(k+1), and $H\left(k+1\right)\in R^{n_{y}\times n_{x}}$ is the corresponding measurement matrix. The process noise w(k) and measurement noise v(k+1) are assumed to be zero-mean Gaussian white-noise sequences satisfying

(2) $$E\left\{w\left(k\right)\right\}=0,E\left\{w\left(k\right)w^{T}\left(j\right)\right\}=Q\left(k\right)\delta_{kj},$$

(3) $$E\left\{v\left(k+1\right)\right\}=0,E\left\{v\left(k+1\right)v^{T}\left(j+1\right)\right\}=R\left(k+1\right)\delta_{kj},$$

and $E\{w\left(k\right)v^{T}\left(j+1\right)\}=0$, where $Q\left(k\right)\in R^{n_{x}\times n_{x}}$ and $R\left(k+1\right)\in R^{n_{y}\times n_{y}}$ denote the covariance matrices of the process noise and measurement noise, respectively, and where $\delta_{kj}$ denotes the Kronecker delta, defined as $\delta_{kj}=\left\{\begin{array}{cc} 1, & k=j,\\ 0, & k\neq j, \end{array}\right.$

which characterizes the temporal whiteness of the noise sequences. Specifically, the covariance is nonzero only when the two noise samples correspond to the same time instant, whereas noise samples at different time instants are uncorrelated.

Building on this linear-system model, the proposed PEPKF employs Kronecker augmentation to extend the prediction into second-, third-, and higher-order polynomial representation spaces without modifying the physical system or measurement model. The enlarged prediction space provides an explicit representation of higher-order state-coupling and predictive statistical structures for subsequent fusion.

## 3. Preliminaries on the Kronecker Product

The Kronecker product offers computational advantages when dealing with high-dimensional problems, as it enables high-dimensional information to be extracted and processed using low-dimensional techniques. In this work, the Kronecker product is employed to augment the dimensionality of the model equations. The key properties of the Kronecker product required in the subsequent derivations are briefly summarized below.

**Definition 1.**  *For any integer h≥0 and arbitrary $a,b\in R^{n}$, the following relation holds [26,27]:*

(4) $${\left(a+b\right)}^{\left[h\right]}=\sum_{k=0}^{h}M_{h}^{k}\left(a^{\left[k\right]}⊗b^{\left[h-k\right]}\right).$$

**Property 1.**  *For $a\in R^{m\times 1}$ and $b\in R^{p\times 1}$, one has*

(5) $$a⊗b=M\left[b⊗a\right].$$

*In Equation (5), the vector b⊗a is obtained by rearranging the elements of a⊗b. Therefore, $M\in R^{mp\times mp}$ is a permutation matrix.*

**Property 2.**  *For vectors $a\in R^{m\times 1}$ and $b\in R^{p\times 1}$, the following identity holds:*

(6) $$a⊗b=G_{b}\left(a\right)b.$$

*Here,*

(7) $$G_{b}\left(a\right)b={\left[{\left(a_{1}I_{b}\right)}^{T},\ldots ,{\left(a_{i}I_{b}\right)}^{T},\ldots ,{\left(a_{m}I_{b}\right)}^{T}\right]}^{T},$$

*where $I_{b}\in R^{n\times n}$ denotes the identity matrix.*

## 4. Design of the Prediction-Enhanced Polynomial Kalman Filter (PEPKF)

Built upon the conventional KF prediction, the proposed PEPKF enlarges the candidate prediction space by constructing second-, third-, and higher-order polynomial representations through Kronecker augmentation. Although the physical system model is linear, the predictive transformation itself is nonlinear. For example, the second-order mapping contains $x_{i}^{2}$ and $x_{i}x_{j}$, and it therefore explicitly introduces second-order polynomial state characteristics into the internal prediction process. The resulting higher-order predictive representations are formulated as equivalent pseudo-observations and fused with the original one-step prediction through weighted least squares to obtain a fused prior estimate. The standard Kalman measurement-update structure is then retained to complete the state estimation.

### 4.1. Kalman Filter State Estimation and Error Characterization

Let x(k) denote the true state of the system at time *k*. Using all measurement information available up to time *k*, i.e., {y(1),y(2),…,y(k)}, the Kalman filtering estimate of the system state is denoted by $\hat{x}\left(k|k\right)$, where $\hat{x}\left(k|k\right)$ represents the optimal estimate of the true state x(k) in the minimum mean-square-error sense. The corresponding state-estimation error is defined as $\tilde{x}\left(k|k\right)=x\left(k\right)-\hat{x}\left(k|k\right)$, and its estimation-error covariance matrix is defined as $P\left(k|k\right)=E\left\{\tilde{x}\left(k|k\right)x{\left(k|k\right)}^{T}\right\}$.

Based on the state estimate at time *k*, the one-step-ahead prediction $\hat{x}\left(k+1|k\right)$ can be obtained. Its prediction error is defined as $\tilde{x}\left(k+1|k\right)=x\left(k+1\right)-\hat{x}\left(k+1|k\right)$, and the corresponding prediction-error covariance matrix is $P\left(k+1|k\right)=E\left\{\tilde{x}\left(k+1|k\right)x{\left(k+1|k\right)}^{T}\right\}$.

### 4.2. Generation of an Enhanced State-Prediction Representation

In this paper, $n_{x}$ and $n_{y}$ denote the dimensions of the state and measurement vectors, respectively. The matrices *A*, *H*, *Q*, and *R* denote the state-transition matrix, measurement matrix, process-noise covariance matrix, and measurement-noise covariance matrix, respectively. The superscript [r] denotes the *r*th-order Kronecker representation, and ⊗ denotes the Kronecker product.

From

(8) $$\begin{array}{c} x\left(k+1\right)=\hat{x}\left(k+1|k\right)+\tilde{x}\left(k+1|k\right), \end{array}$$

a second-order Kronecker expansion is applied to both sides of Equation (8) [28], yielding

(9) $$\begin{array}{c} {\left[\hat{x}\left(k+1|k\right)+\tilde{x}\left(k+1|k\right)\right]}^{\left[2\right]}=x^{\left[2\right]}\left(k+1\right). \end{array}$$

The left-hand side can be expanded as

(10) $$\begin{array}{cc} \; & {\left[\hat{x}\left(k+1|k\right)+\tilde{x}\left(k+1|k\right)\right]}^{\left[2\right]}\\ \; & ={\hat{x}}^{\left[2\right]}\left(k+1|k\right)+\hat{x}\left(k+1|k\right)⊗\tilde{x}\left(k+1|k\right)+M\left[\hat{x}\left(k+1|k\right)⊗x\left(k+1|k\right)\right]\\ \; & \;+{\hat{\tilde{x}}}^{\left[2\right]}\left(k+1|k\right)+{\tilde{\tilde{x}}}^{\left[2\right]}\left(k+1|k\right)\\ \; & ={\hat{x}}^{\left[2\right]}\left(k+1|k\right)+G\left\{\hat{x}\left(k+1|k\right)\right\}\tilde{x}\left(k+1|k\right)+M\left[G\{\hat{x}\left(k+1|k\right)\}x\left(k+1|k\right)\right]\\ \; & \;+{\hat{\tilde{x}}}^{\left[2\right]}\left(k+1|k\right)+{\tilde{\tilde{x}}}^{\left[2\right]}\\ \; & ={\hat{x}}^{\left[2\right]}\left(k+1|k\right)+B^{\left[2,1\right]}\left(k\right)\tilde{x}\left(k+1|k\right)+B^{\left[2,2\right]}\left(k\right){\hat{\tilde{x}}}^{\left[2\right]}\left(k+1|k\right)+B^{\left[2,2\right]}\left(k\right){\tilde{\tilde{x}}}^{\left[2\right]}. \end{array}$$

**Remark 1.**  *Here, M is a permutation matrix,* ⊗ *denotes the Kronecker product, and $x^{\left[2\right]}$ represents the second-order polynomial state representation.*

(11) $$B^{\left[2,1\right]}\left(k\right)=\left(I+M\right)G\left\{\hat{x}\left(k+1|k\right)\right\},\;B^{\left[2,2\right]}\left(k\right)=I.$$

Furthermore, it should be noted that the exact second-order state representation contains the term x(k+1)⊗x(k+1). In order to construct a linear pseudo-observation form with respect to the predicted state, the following approximation is adopted:

(12) $$\begin{array}{cc} x^{\left[2\right]}\left(k+1\right) & =x(k+1)⊗x(k+1)\\ \; & \approx \hat{x}\left(k+1|k\right)⊗x\left(k+1\right)\\ \; & =G\{\hat{x}\left(k+1|k\right)\}x\left(k+1\right). \end{array}$$

This replacement is a local approximation rather than an exact identity. The associated approximation residual and its possible bias are explicitly characterized in the subsequent general *r*th-order formulation.

Therefore, Equation (9) can be simplified to

(13) $$\begin{array}{c} x^{\left[2,1\right]}\left(k+1\right)=H_{x}^{\left[2,1\right]}\left(k+1\right)x\left(k+1\right)+v_{x}^{\left[2,1\right]}\left(k+1\right), \end{array}$$

where

(14) $$\begin{array}{cc} x^{\left[2,1\right]}\left(k+1\right) & ={\hat{x}}^{\left[2\right]}\left(k+1|k\right)+{\hat{\tilde{x}}}^{\left[2\right]}\left(k+1|k\right),\\ H_{x}^{\left[2,1\right]}\left(k+1\right) & =G\{\hat{x}\left(k+1|k\right)\},\\ v_{x}^{\left[2,1\right]}\left(k+1\right) & =-B^{\left[2,1\right]}\left(k\right)\tilde{x}\left(k+1|k\right)-B^{\left[2,2\right]}\left(k\right){\tilde{\tilde{x}}}^{\left[2\right]}\left(k+1|k\right). \end{array}$$

The second-order prediction-error moments are approximated using the scaled sigma-point rule. Let the dimension of the one-step prediction error be *n*. For the zero-mean prediction error

(15) $$\tilde{x}\left(k+1|k\right)∼N\left(0,P(k+1|k)\right),$$

a set of 2n+1 sigma points is constructed as

(16) $$\begin{array}{cc} X_{0}\left(k+1|k\right) & =0,\\ X_{i}\left(k+1|k\right) & ={\left[\sqrt{\left(n+\lambda )P(k+1|k\right)}\right]}_{i},\;i=1,\ldots ,n,\\ X_{i+n}\left(k+1|k\right) & =-{\left[\sqrt{\left(n+\lambda )P(k+1|k\right)}\right]}_{i},\;i=1,\ldots ,n, \end{array}$$

where

(17) $$\lambda =\alpha^{2}\left(n+\kappa \right)-n.$$

Here, α and κ are scaling parameters that control the spread of the sigma points. The corresponding weights for the mean and covariance are defined as

(18) $$\begin{array}{cc} W_{0}^{\left(m\right)} & =\frac{\lambda }{n+\lambda },\\ W_{0}^{\left(c\right)} & =\frac{\lambda }{n+\lambda }+\left(1-\alpha^{2}+\beta \right),\\ W_{i}^{\left(m\right)} & =W_{i}^{\left(c\right)}=\frac{1}{2(n+\lambda )},\;i=1,\ldots ,2n, \end{array}$$

where β incorporates prior information about the distribution of the prediction error.

For each sigma point $X_{i}\left(k+1|k\right)$, define the corresponding second-order Kronecker mapping as

(19) $$X_{i}^{\left[2\right]}\left(k+1|k\right)=X_{i}\left(k+1|k\right)⊗X_{i}(k+1|k).$$

The mean of the second-order prediction-error representation is approximated by

(20) $$m_{\tilde{x}}^{\left[2\right]}\left(k+1|k\right)\approx \sum_{i=0}^{2n}W_{i}^{\left(m\right)}X_{i}^{\left[2\right]}(k+1|k).$$

The second-order Kronecker prediction error is defined as

(21) $${\tilde{x}}^{\left[2\right]}\left(k+1|k\right)=\tilde{x}\left(k+1|k\right)⊗\tilde{x}(k+1|k).$$

Accordingly, the centered second-order prediction error is defined as

(22) $${\tilde{\tilde{x}}}^{\left[2\right]}\left(k+1|k\right)={\tilde{x}}^{\left[2\right]}\left(k+1|k\right)-m_{\tilde{x}}^{\left[2\right]}(k+1|k).$$

By construction,

(23) $$E\left\{{\tilde{\tilde{x}}}^{\left[2\right]}\left(k+1|k\right)\right\}=0.$$

The covariance of the centered second-order prediction error is approximated as

(24) $$\begin{array}{cc} P_{\tilde{x}\tilde{x}}^{\left[2,2\right]}\left(k+1|k\right) & \approx \sum_{i=0}^{2n}W_{i}^{\left(c\right)}\left[X_{i}^{\left[2\right]}\left(k+1|k\right)-m_{\tilde{x}}^{\left[2\right]}\left(k+1|k\right)\right]\\ \; & \;\times {\left[X_{i}^{\left[2\right]}\left(k+1|k\right)-m_{\tilde{x}}^{\left[2\right]}\left(k+1|k\right)\right]}^{T}. \end{array}$$

In addition to the second-order autocovariance, the cross-covariance between the first-order prediction error and the centered second-order prediction error is defined as

(25) $$P_{\tilde{x}\tilde{x}}^{\left[1,2\right]}\left(k+1|k\right)=E\left\{\tilde{x}\left(k+1|k\right){\tilde{\tilde{x}}}^{[2]T}\left(k+1|k\right)\right\}.$$

Using the same sigma-point set, this cross-covariance can be approximated by

(26) $$\begin{array}{cc} P_{\tilde{x}\tilde{x}}^{\left[1,2\right]}\left(k+1|k\right) & \approx \sum_{i=0}^{2n}W_{i}^{\left(c\right)}X_{i}\left(k+1|k\right)\\ \; & \;\times {\left[X_{i}^{\left[2\right]}\left(k+1|k\right)-m_{\tilde{x}}^{\left[2\right]}\left(k+1|k\right)\right]}^{T}. \end{array}$$

Similarly,

(27) $$P_{\tilde{x}\tilde{x}}^{\left[2,1\right]}\left(k+1|k\right)=P_{\tilde{x}\tilde{x}}^{[1,2]T}(k+1|k).$$

Although ${\tilde{\tilde{x}}}^{\left[2\right]}\left(k+1|k\right)$ is a second-order Kronecker representation, its cross-covariance with the first-order prediction error involves third-order central moments. Under the zero-mean Gaussian assumption for $\tilde{x}\left(k+1|k\right)$, all third-order central moments vanish. Therefore,

(28) $$P_{\tilde{x}\tilde{x}}^{\left[1,2\right]}\left(k+1|k\right)=P_{\tilde{x}\tilde{x}}^{\left[2,1\right]}\left(k+1|k\right)=0.$$

The covariance matrix of the second-order pseudo-observation error is defined as

(29) $$R_{x}^{\left[2,1\right]}\left(k+1\right)=E\left\{v_{x}^{\left[2,1\right]}\left(k+1\right)v_{x}^{[2,1]T}\left(k+1\right)\right\}.$$

According to

(30) $$v_{x}^{\left[2,1\right]}\left(k+1\right)=-B^{\left[2,1\right]}\left(k\right)\tilde{x}\left(k+1|k\right)-B^{\left[2,2\right]}\left(k\right){\tilde{\tilde{x}}}^{\left[2\right]}(k+1|k),$$

the complete covariance expression is

(31) $$\begin{array}{cc} R_{x}^{\left[2,1\right]}\left(k+1\right) & =B^{\left[2,1\right]}\left(k\right)P\left(k+1|k\right)B^{[2,1]T}\left(k\right)\\ \; & \;+B^{\left[2,2\right]}\left(k\right)P_{\tilde{x}\tilde{x}}^{\left[2,2\right]}\left(k+1|k\right)B^{[2,2]T}\left(k\right)\\ \; & \;+B^{\left[2,1\right]}\left(k\right)P_{\tilde{x}\tilde{x}}^{\left[1,2\right]}\left(k+1|k\right)B^{[2,2]T}\left(k\right)\\ \; & \;+B^{\left[2,2\right]}\left(k\right)P_{\tilde{x}\tilde{x}}^{\left[2,1\right]}\left(k+1|k\right)B^{[2,1]T}(k). \end{array}$$

Using Equation (28), the first-order–second-order cross terms vanish under the zero-mean Gaussian condition. Therefore, the covariance matrix of the second-order pseudo-observation error reduces to

(32) $$\begin{array}{cc} R_{x}^{\left[2,1\right]}\left(k+1\right) & =B^{\left[2,1\right]}\left(k\right)P\left(k+1|k\right)B^{[2,1]T}\left(k\right)\\ \; & \;+B^{\left[2,2\right]}\left(k\right)P_{\tilde{x}\tilde{x}}^{\left[2,2\right]}\left(k+1|k\right)B^{[2,2]T}(k). \end{array}$$

The zero cross-covariance in Equation (28) only applies between the first-order prediction error and the centered second-order prediction error under the zero-mean Gaussian condition. The $v_{x}^{\left[2,1\right]}\left(k+1\right)$ contains a first-order prediction-error component, so the correlation between the original one-step prediction error and the complete second-order pseudo-observation error is explicitly removed through the orthogonalization procedure before the subsequent weighted least-squares fusion.

Combining Equations (8) and (13), the second-order augmented model of the system equation is obtained as

(33) $$\begin{array}{c} {\bar{x}}^{\left(2\right)}\left(k+1|k\right)={\bar{H}}_{x}^{\left(2\right)}\left(k+1\right)x\left(k+1\right)+{\bar{v}}_{x}^{\left(2\right)}\left(k+1\right), \end{array}$$

where

(34) $$\begin{array}{cc} {\bar{x}}^{\left(2\right)}\left(k+1|k\right) & ={\left[\hat{x}\left(k+1|k\right),x^{\left[2,1\right]}\left(k+1\right)\right]}^{T},\\ {\bar{H}}_{x}^{\left(2\right)}\left(k+1\right) & ={\left[I,H_{x}^{\left[2,1\right]}\left(k+1\right)\right]}^{T},\\ {\bar{v}}_{x}^{\left(2\right)}\left(k+1\right) & ={\left[-\tilde{x}\left(k+1|k\right),v_{x}^{\left[2,1\right]}\left(k+1\right)\right]}^{T},\\ {\bar{R}}^{\left(2\right)} & =\left[R,R^{\left(2,1\right)}\right]I_{2\times 2}. \end{array}$$

Because the original one-step prediction error and the second-order pseudo-observation error are generally correlated, the two predictive representations cannot be fused directly. Their cross-covariance is first evaluated, followed by a Gram–Schmidt orthogonalization to remove the correlated component. Weighted least squares is then applied to the resulting orthogonalized joint predictive model. The complete derivation is provided in Appendix A.

The resulting second-order fused prediction covariance can be expressed as

(35) $${\bar{P}}^{\left(2\right)}\left(k+1|k\right)={\left[P^{-1}\left(k+1|k\right)+{\bar{H}}_{2}^{T}\left(k+1\right){\bar{R}}_{2}^{-1}\left(k+1\right){\bar{H}}_{2}\left(k+1\right)\right]}^{-1},$$

where ${\bar{H}}_{2}\left(k+1\right)$ and ${\bar{R}}_{2}\left(k+1\right)$ denote the equivalent observation matrix and error covariance after orthogonalization, where the superscript (2) is used to denotes the fused state prediction obtained from the first- and second-order predictive representations.

The resulting second-order fused prediction obtained from the orthogonalized weighted least-squares model is

(36) $$\begin{array}{cc} {\hat{\bar{x}}}^{\left(2\right)}\left(k+1|k\right) & ={\left[{\bar{H}}_{x}^{(2)T}\left(k+1\right){\bar{R}}_{x}^{(2)-1}\left(k+1\right){\bar{H}}_{x}^{\left(2\right)}\left(k+1\right)\right]}^{-1}\\ \; & \;\times {\bar{H}}_{x}^{(2)T}\left(k+1\right){\bar{R}}_{x}^{(2)-1}\left(k+1\right){\bar{x}}^{\left(2\right)}(k+1). \end{array}$$

The corresponding conditional identification error is

(37) $$\begin{array}{cc} {\tilde{\bar{x}}}^{\left(2\right)}\left(k+1|k\right) & =x\left(k+1\right)-{\hat{\bar{x}}}^{\left(2\right)}\left(k+1|k\right)\\ \; & =-{\left({\bar{H}}_{x}^{\left(2\right)}{\left(k+1\right)}^{T}{\bar{R}}_{x}^{\left(2\right)}{\left(k+1\right)}^{-1}{\bar{H}}_{x}^{\left(2\right)}\left(k+1\right)\right)}^{-1}\\ \; & \;\times {\bar{H}}_{x}^{\left(2\right)}{\left(k+1\right)}^{T}{\bar{R}}_{x}^{\left(2\right)}{\left(k+1\right)}^{-1}{\bar{v}}_{x}^{\left(2\right)}(k+1). \end{array}$$

### 4.3. Kalman Filtering Recursion Based on Predictive Fusion

Based on

(38) $$\begin{array}{c} \left\{\begin{array}{cc} x(k+1) & ∼N\left[{\hat{x}}^{\left(2\right)}\left(k+1|k\right),P^{\left(2\right)}\left(k+1|k\right)\right],\\ x(k+1) & ∼N\left[\hat{x}\left(k+1|k\right),P\left(k+1|k\right)\right], \end{array}\right. \end{array}$$

the measurement equation is

(39) $$\begin{array}{c} y(k+1)=H(k+1)x(k+1)+v(k+1). \end{array}$$

(1) Measurement-prediction estimate and measurement-prediction error.

The measurement-prediction estimate is

(40) $$\begin{array}{c} \hat{y}\left(k+1|k\right)=H\left(k+1\right){\hat{x}}^{\left(2\right)}\left(k+1|k\right), \end{array}$$

and the measurement-prediction error is

(41) $$\begin{array}{c} \tilde{y}\left(k+1|k\right)=H\left(k+1\right){\bar{\tilde{x}}}^{\left(2\right)}\left(k+1|k\right)+v\left(k+1\right). \end{array}$$

(2) Cross-covariance and autocovariance matrices.

The autocovariance matrix of the measurement-prediction error is

(42) $$\begin{array}{cc} P_{\tilde{y}\tilde{y}}^{\left(2\right)}\left(k+1|k\right) & =E\left\{\tilde{y}\left(k+1|k\right)y{\left(k+1|k\right)}^{T}\right\}\\ \; & =H\left(k+1\right){\bar{P}}^{\left(2\right)}\left(k+1|k\right)H{\left(k+1\right)}^{T}+R\left(k+1\right), \end{array}$$

and the cross-covariance matrix between the state-prediction error and the measurement-prediction error is

(43) $$\begin{array}{cc} P_{\tilde{x}\tilde{y}}^{\left(2\right)}\left(k+1|k\right) & =E\left\{{\bar{\tilde{x}}}^{\left(2\right)}\left(k+1|k\right)\tilde{y}{\left(k+1|k\right)}^{T}\right\}\\ \; & ={\bar{P}}^{\left(2\right)}\left(k+1|k\right)H{\left(k+1\right)}^{T}. \end{array}$$

(3) Polynomial Kalman filter design.

Let $K^{\left(2\right)}\left(k+1\right)$ denote the Kalman gain matrix of the augmented system. Based on the statistical orthogonality principle between the linear minimum-variance state-estimation error and the measurement vector,

(44) $$\begin{array}{c} E\left\{{\tilde{x}}^{\left(2\right)}\left(k+1|k+1\right)\tilde{y}{\left(k+1\right)}^{T}\right\}=0, \end{array}$$

the Kalman gain can be obtained as

(45) $$\begin{array}{c} K^{\left(2\right)}\left(k+1\right)=P_{\tilde{x}\tilde{y}}^{\left(2\right)}\left(k+1|k\right)P_{\tilde{y}\tilde{y}}^{\left(2\right)}{\left(k+1|k\right)}^{-1}. \end{array}$$

The state estimate is updated according to

(46) $$\begin{array}{cc} {\hat{x}}^{\left(2\right)}\left(k+1|k+1\right) & ={\hat{x}}^{\left(2\right)}\left(k+1|k\right)+K^{\left(2\right)}\left(k+1\right)\tilde{y}\left(k+1|k\right)\\ \; & =\left[I-K^{\left(2\right)}\left(k+1\right)H\left(k+1\right)\right]{\hat{x}}^{\left(2\right)}\left(k+1|k\right)\\ \; & \;+K^{\left(2\right)}\left(k+1\right)y\left(k+1\right). \end{array}$$

The corresponding estimation error is

(47) $$\begin{array}{cc} {\tilde{x}}^{\left(2\right)}\left(k+1|k+1\right) & =x\left(k+1\right)-{\hat{x}}^{\left(2\right)}\left(k+1|k+1\right)\\ \; & ={\bar{\tilde{x}}}^{\left(2\right)}\left(k+1|k\right)-K^{\left(2\right)}\left(k+1\right)\left[H\left(k+1\right)x^{\left(2\right)}\left(k+1|k\right)+v\left(k+1\right)\right]. \end{array}$$

The estimation-error covariance matrix is

(48) $$\begin{array}{cc} P_{\tilde{x}\tilde{x}}^{\left(2\right)}\left(k+1|k+1\right) & =E\left\{{\tilde{x}}^{\left(2\right)}\left(k+1|k+1\right)x^{\left(2\right)}{\left(k+1|k+1\right)}^{T}\right\}\\ \; & =\left[I-K^{\left(2\right)}\left(k+1\right)H\left(k+1\right)\right]{\bar{P}}^{\left(2\right)}\left(k+1|k\right). \end{array}$$

The above derivation gives the recursive form of the second-order prediction-enhanced polynomial Kalman filter. Furthermore, by applying an *r*th-order Kronecker expansion to the one-step predicted state and the corresponding prediction error, where r=2,…,n, the second-order formulation can be generalized to an *n*th-order PEPKF.

The detailed derivation of the proposed *n*th-order prediction-enhanced polynomial Kalman filter is given below.

### 4.4. Generation of State-Prediction Representations up to the nth Order

Let the state dimension be denoted by *d*, the highest polynomial augmentation order by *n*, and an arbitrary intermediate order by *r*, where r=2,…,n.

The true state at time k+1 is expressed as

(49) $$\begin{array}{c} x\left(k+1\right)=\hat{x}\left(k+1|k\right)+\tilde{x}\left(k+1|k\right), \end{array}$$

where $\hat{x}\left(k+1|k\right)\in R^{d}$ denotes the one-step state prediction and $\tilde{x}\left(k+1|k\right)\in R^{d}$ denotes the corresponding prediction error. The prediction-error statistics satisfy

(50) $$\begin{array}{cc} E\left\{\tilde{x}\left(k+1|k\right)\right\} & =0,\\ P(k+1|k) & =E\left\{\tilde{x}\left(k+1|k\right){\tilde{x}}^{T}\left(k+1|k\right)\right\}. \end{array}$$

For any positive integer *r*, define the *r*th-order Kronecker power of a vector *a* as

(51) $$\begin{array}{c} a^{\left[r\right]}=\underset{r\;\mathrm{times}}{\underset{︸}{a⊗a⊗\ldots ⊗a}},\;a^{\left[0\right]}=1. \end{array}$$

Applying the *r*th-order Kronecker expansion to Equation (49) gives

(52) $$\begin{array}{c} x^{\left[r\right]}\left(k+1\right)={\left[\hat{x}\left(k+1|k\right)+\tilde{x}\left(k+1|k\right)\right]}^{\left[r\right]}. \end{array}$$

Using the polynomial expansion property of the Kronecker product, Equation (52) can be written as

(53) $$\begin{array}{c} x^{\left[r\right]}\left(k+1\right)=\sum_{j=0}^{r}M_{r}^{j}\left[{\hat{x}}^{\left[r-j\right]}\left(k+1|k\right)⊗{\tilde{x}}^{\left[j\right]}\left(k+1|k\right)\right], \end{array}$$

where $M_{r}^{j}\in R^{d^{r}\times d^{r}}$ is a combination matrix formed by the sum of all distinct permutation matrices that distribute *j* prediction-error factors among the *r* Kronecker positions. The number of distinct permutation terms is rj, and

(54) $$\begin{array}{c} M_{r}^{0}=M_{r}^{r}=I_{d^{r}}. \end{array}$$

### 4.5. Higher-Order Prediction-Error Statistics

For an arbitrary positive integer *j*, define

(55) $$\begin{array}{c} {\tilde{x}}^{\left[j\right]}\left(k+1|k\right)=\underset{j\;\mathrm{times}}{\underset{︸}{\tilde{x}\left(k+1|k\right)⊗\ldots ⊗\tilde{x}\left(k+1|k\right)}}. \end{array}$$

Although the first-order prediction error is zero mean, its higher-order Kronecker powers generally have nonzero means. Therefore, define

(56) $$\begin{array}{c} m_{\tilde{x}}^{\left[j\right]}\left(k+1|k\right)=E\left\{{\tilde{x}}^{\left[j\right]}\left(k+1|k\right)\right\}. \end{array}$$

The centered *j*th-order prediction error is defined as

(57) $$\begin{array}{c} {\tilde{\tilde{x}}}^{\left[j\right]}\left(k+1|k\right)={\tilde{x}}^{\left[j\right]}\left(k+1|k\right)-m_{\tilde{x}}^{\left[j\right]}\left(k+1|k\right), \end{array}$$

which satisfies

(58) $$\begin{array}{c} E\left\{{\tilde{\tilde{x}}}^{\left[j\right]}\left(k+1|k\right)\right\}=0. \end{array}$$

For the first-order term,

(59) $$\begin{array}{cc} m_{\tilde{x}}^{\left[1\right]}\left(k+1|k\right) & =0,\\ {\tilde{\tilde{x}}}^{\left[1\right]}\left(k+1|k\right) & =\tilde{x}\left(k+1|k\right). \end{array}$$

For any two orders *j* and *ℓ*, define the cross-covariance between the corresponding centered prediction errors as

(60) $$\begin{array}{c} P_{\tilde{x}\tilde{x}}^{\left[j,\ell \right]}\left(k+1|k\right)=E\left\{{\tilde{\tilde{x}}}^{\left[j\right]}\left(k+1|k\right){\tilde{\tilde{x}}}^{[\ell ]T}\left(k+1|k\right)\right\}, \end{array}$$

where

(61) $$\begin{array}{c} P_{\tilde{x}\tilde{x}}^{\left[j,\ell \right]}\left(k+1|k\right)\in R^{d^{j}\times d^{\ell }}. \end{array}$$

The cross-covariances satisfy

(62) $$\begin{array}{c} P_{\tilde{x}\tilde{x}}^{\left[\ell ,j\right]}\left(k+1|k\right)=P_{\tilde{x}\tilde{x}}^{[j,\ell ]T}\left(k+1|k\right). \end{array}$$

When j=ℓ, Equation (60) reduces to

(63) $$\begin{array}{c} P_{\tilde{x}\tilde{x}}^{\left[j,j\right]}\left(k+1|k\right)=E\left\{{\tilde{\tilde{x}}}^{\left[j\right]}\left(k+1|k\right){\tilde{\tilde{x}}}^{[j]T}\left(k+1|k\right)\right\}. \end{array}$$

In particular,

(64) $$\begin{array}{c} P_{\tilde{x}\tilde{x}}^{\left[1,1\right]}\left(k+1|k\right)=P\left(k+1|k\right). \end{array}$$

### 4.6. Sigma-Point-Based Computation of Higher-Order Error Moments

Let

(65) $$\begin{array}{c} X_{i}\left(k+1|k\right),\;i=0,1,\ldots ,2d, \end{array}$$

denote the 2d+1 sigma points generated from the zero-mean prediction-error distribution with covariance P(k+1|k).

Mapping the *i*th sigma point into the *j*th-order Kronecker space gives

(66) $$\begin{array}{c} X_{i}^{\left[j\right]}\left(k+1|k\right)=\underset{j\;\mathrm{times}}{\underset{︸}{X_{i}\left(k+1|k\right)⊗\ldots ⊗X_{i}\left(k+1|k\right)}}. \end{array}$$

The *j*th-order prediction-error moment is approximated as

(67) $$\begin{array}{c} m_{\tilde{x}}^{\left[j\right]}\left(k+1|k\right)\approx \sum_{i=0}^{2d}W_{i}^{\left(m\right)}X_{i}^{\left[j\right]}\left(k+1|k\right). \end{array}$$

Define the centered *j*th-order sigma-point representation as

(68) $$\begin{array}{c} \delta X_{i}^{\left[j\right]}\left(k+1|k\right)=X_{i}^{\left[j\right]}\left(k+1|k\right)-m_{\tilde{x}}^{\left[j\right]}\left(k+1|k\right). \end{array}$$

The cross-covariance between the *j*th- and *ℓ*th-order centered prediction errors is approximated by

(69) $$\begin{array}{c} P_{\tilde{x}\tilde{x}}^{\left[j,\ell \right]}\left(k+1|k\right)\approx \sum_{i=0}^{2d}W_{i}^{\left(c\right)}\delta X_{i}^{\left[j\right]}\left(k+1|k\right)\delta X_{i}^{[\ell ]T}\left(k+1|k\right). \end{array}$$

When j=ℓ, one obtains

(70) $$\begin{array}{c} P_{\tilde{x}\tilde{x}}^{\left[j,j\right]}\left(k+1|k\right)\approx \sum_{i=0}^{2d}W_{i}^{\left(c\right)}\delta X_{i}^{\left[j\right]}\left(k+1|k\right)\delta X_{i}^{[j]T}\left(k+1|k\right). \end{array}$$

### 4.7. The rth-Order Polynomial Pseudo-Observation Model

To simplify Equation (53), define

(71) $$\begin{array}{c} B^{\left[r,j\right]}\left(k\right)=M_{r}^{j}\left[{\hat{x}}^{\left[r-j\right]}\left(k+1|k\right)⊗I_{d^{j}}\right],\;j=1,\ldots ,r, \end{array}$$

where

(72) $$\begin{array}{c} B^{\left[r,j\right]}\left(k\right)\in R^{d^{r}\times d^{j}}. \end{array}$$

Thus,

(73) $$\begin{array}{c} M_{r}^{j}\left[{\hat{x}}^{\left[r-j\right]}\left(k+1|k\right)⊗{\tilde{x}}^{\left[j\right]}\left(k+1|k\right)\right]=B^{\left[r,j\right]}\left(k\right){\tilde{x}}^{\left[j\right]}\left(k+1|k\right). \end{array}$$

Using

(74) $$\begin{array}{c} {\tilde{x}}^{\left[j\right]}\left(k+1|k\right)=m_{\tilde{x}}^{\left[j\right]}\left(k+1|k\right)+{\tilde{\tilde{x}}}^{\left[j\right]}\left(k+1|k\right), \end{array}$$

Equation (53) becomes

(75) $$\begin{array}{cc} x^{\left[r\right]}\left(k+1\right) & ={\hat{x}}^{\left[r\right]}\left(k+1|k\right)+\sum_{j=1}^{r}B^{\left[r,j\right]}\left(k\right)m_{\tilde{x}}^{\left[j\right]}\left(k+1|k\right)\\ \; & \;+\sum_{j=1}^{r}B^{\left[r,j\right]}\left(k\right){\tilde{\tilde{x}}}^{\left[j\right]}\left(k+1|k\right). \end{array}$$

Since $m_{\tilde{x}}^{\left[1\right]}\left(k+1|k\right)=0$, define the *r*th-order polynomial prediction quantity as

(76) $$\begin{array}{cc} {\hat{x}}^{\left[r,1\right]}\left(k+1|k\right) & ={\hat{x}}^{\left[r\right]}\left(k+1|k\right)\\ \; & \;+\sum_{j=2}^{r}B^{\left[r,j\right]}\left(k\right)m_{\tilde{x}}^{\left[j\right]}\left(k+1|k\right). \end{array}$$

Consistent with the second-order construction, introduce the local linear representation

(77) $$\begin{array}{c} x^{\left[r\right]}\left(k+1\right)\approx H_{x}^{\left[r,1\right]}\left(k+1\right)x\left(k+1\right), \end{array}$$

where

(78) $$H_{x}^{\left[r,1\right]}\left(k+1\right)={\hat{x}}^{\left[r-1\right]}\left(k+1|k\right)⊗I_{d}.$$

The approximation residual associated with Equation (77) is defined as

(79) $$\begin{array}{c} \rho_{r}\left(k+1\right)=x^{\left[r\right]}\left(k+1\right)-H_{x}^{\left[r,1\right]}\left(k+1\right)x\left(k+1\right). \end{array}$$

Combining Equations (75) and (79), the *r*th-order polynomial pseudo-observation can be written as

(80) $$\begin{array}{c} {\hat{x}}^{\left[r,1\right]}\left(k+1|k\right)=H_{x}^{\left[r,1\right]}\left(k+1\right)x\left(k+1\right)+v_{x}^{\left[r,1\right]}\left(k+1\right), \end{array}$$

where the equivalent pseudo-observation error is

(81) $$v_{x}^{\left[r,1\right]}\left(k+1\right)=\rho_{r}\left(k+1\right)-\sum_{j=1}^{r}B^{\left[r,j\right]}\left(k\right){\tilde{\tilde{x}}}^{\left[j\right]}\left(k+1|k\right).$$

Since the approximation residual $\rho_{r}\left(k+1\right)$ is not necessarily zero mean, define its mean as

(82) $$b_{r}\left(k+1\right)=E\left\{\rho_{r}\left(k+1\right)\right\}.$$

The corresponding centered residual is

(83) $$\rho_{r}^{c}\left(k+1\right)=\rho_{r}\left(k+1\right)-b_{r}\left(k+1\right),$$

which satisfies

(84) $$E\left\{\rho_{r}^{c}\left(k+1\right)\right\}=0.$$

Accordingly, define the bias-corrected pseudo-observation as

(85) $${\hat{x}}_{c}^{\left[r,1\right]}\left(k+1|k\right)={\hat{x}}^{\left[r,1\right]}\left(k+1|k\right)-b_{r}\left(k+1\right).$$

The corresponding equivalent error becomes

(86) $$v_{x,c}^{\left[r,1\right]}\left(k+1\right)=\rho_{r}^{c}\left(k+1\right)-\sum_{j=1}^{r}B^{\left[r,j\right]}\left(k\right){\tilde{\tilde{x}}}^{\left[j\right]}\left(k+1|k\right),$$

so that

(87) $$E\left\{v_{x,c}^{\left[r,1\right]}\left(k+1\right)\right\}=0,$$

provided that the centered higher-order prediction-error quantities are used.

Therefore, a possible bias caused by the local polynomial approximation is explicitly separated from the zero-mean stochastic error. When the local approximation residual is sufficiently small, $b_{r}\left(k+1\right)$ and $\rho_{r}^{c}\left(k+1\right)$ may be neglected, yielding the residual-free form used in the subsequent predictive-fusion derivation.

When the local approximation residual is negligible, Equation (81) reduces to

(88) $$\begin{array}{c} v_{x}^{\left[r,1\right]}\left(k+1\right)\approx -\sum_{j=1}^{r}B^{\left[r,j\right]}\left(k\right){\tilde{\tilde{x}}}^{\left[j\right]}\left(k+1|k\right). \end{array}$$

### 4.8. Covariance and Cross-Covariance of the Pseudo-Observation Errors

For the residual-neglected form in Equation (88), the complete covariance of the *r*th-order pseudo-observation error is

(89) $$\begin{array}{cc} R_{x}^{\left[r,1\right]}\left(k+1\right) & =E\left\{v_{x}^{\left[r,1\right]}\left(k+1\right)v_{x}^{[r,1]T}\left(k+1\right)\right\}\\ \; & \approx \sum_{j=1}^{r}\sum_{\ell =1}^{r}B^{\left[r,j\right]}\left(k\right)P_{\tilde{x}\tilde{x}}^{\left[j,\ell \right]}\left(k+1|k\right)B^{[r,\ell ]T}\left(k\right). \end{array}$$

Equation (89) retains both the same-order covariance terms and the cross-order covariance terms.

For two pseudo-observation orders *r* and *s*, their cross-covariance is defined as

(90) $$\begin{array}{cc} R_{rs}\left(k+1\right) & =E\left\{v_{x}^{\left[r,1\right]}\left(k+1\right)v_{x}^{[s,1]T}\left(k+1\right)\right\}\\ \; & \approx \sum_{j=1}^{r}\sum_{\ell =1}^{s}B^{\left[r,j\right]}\left(k\right)P_{\tilde{x}\tilde{x}}^{\left[j,\ell \right]}\left(k+1|k\right)B^{[s,\ell ]T}\left(k\right). \end{array}$$

The cross-covariance between the *r*th-order pseudo-observation error and the original one-step prediction error $v_{1}\left(k+1\right)=-\tilde{x}\left(k+1|k\right)$ is

(91) $$\begin{array}{cc} R_{r1}\left(k+1\right) & =E\left\{v_{x}^{\left[r,1\right]}\left(k+1\right)v_{1}^{T}\left(k+1\right)\right\}\\ \; & \approx \sum_{j=1}^{r}B^{\left[r,j\right]}\left(k\right)P_{\tilde{x}\tilde{x}}^{\left[j,1\right]}\left(k+1|k\right). \end{array}$$

The above covariance and cross-covariance matrices provide the statistical quantities required for the subsequent orthogonalization.

### 4.9. Sequential Orthogonalization of Multi-Order Predictive Information

Because the original one-step prediction and the higher-order pseudo-observations are constructed from the same underlying prediction-error distribution, their equivalent errors are generally correlated. Therefore, a sequential Gram–Schmidt orthogonalization is applied before the weighted least-squares fusion.

For compact notation, define the first-order predictive representation as

(92) $$\begin{array}{cc} z_{1}\left(k+1\right) & =\hat{x}\left(k+1|k\right),\\ H_{1}\left(k+1\right) & =I_{d},\\ v_{1}\left(k+1\right) & =-\tilde{x}\left(k+1|k\right),\\ R_{1}\left(k+1\right) & =P(k+1|k). \end{array}$$

For r=2,…,n, define

(93) $$\begin{array}{cc} z_{r}\left(k+1\right) & ={\hat{x}}^{\left[r,1\right]}\left(k+1|k\right),\\ H_{r}\left(k+1\right) & =H_{x}^{\left[r,1\right]}\left(k+1\right),\\ v_{r}\left(k+1\right) & =v_{x}^{\left[r,1\right]}\left(k+1\right). \end{array}$$

The first representation is retained unchanged:

(94) $$\begin{array}{cc} {\bar{z}}_{1}\left(k+1\right) & =z_{1}\left(k+1\right),\\ {\bar{H}}_{1}\left(k+1\right) & =H_{1}\left(k+1\right),\\ {\bar{v}}_{1}\left(k+1\right) & =v_{1}\left(k+1\right),\\ {\bar{R}}_{1}\left(k+1\right) & =R_{1}\left(k+1\right). \end{array}$$

For each r=2,…,n, initialize

(95) $$\begin{array}{cc} z_{r}^{\left(0\right)}\left(k+1\right) & =z_{r}\left(k+1\right),\\ H_{r}^{\left(0\right)}\left(k+1\right) & =H_{r}\left(k+1\right),\\ v_{r}^{\left(0\right)}\left(k+1\right) & =v_{r}\left(k+1\right),\\ R_{r}^{\left(0\right)}\left(k+1\right) & =R_{x}^{\left[r,1\right]}\left(k+1\right). \end{array}$$

For j=1,…,r−1, define the cross-covariance between the current *r*th-order residual error and the *j*th orthogonalized error as

(96) $$\begin{array}{c} F_{rj}\left(k+1\right)=E\left\{v_{r}^{\left(j-1\right)}\left(k+1\right){\bar{v}}_{j}^{T}\left(k+1\right)\right\}. \end{array}$$

The corresponding orthogonalization matrix is

(97) $$\begin{array}{c} G_{rj}\left(k+1\right)=F_{rj}\left(k+1\right){\bar{R}}_{j}^{-1}\left(k+1\right). \end{array}$$

The *r*th-order error, pseudo-observation, and observation matrix are updated recursively according to

(98) $$v_{r}^{\left(j\right)}\left(k+1\right)=v_{r}^{\left(j-1\right)}\left(k+1\right)-G_{rj}\left(k+1\right){\bar{v}}_{j}\left(k+1\right),$$

(99) $$z_{r}^{\left(j\right)}\left(k+1\right)=z_{r}^{\left(j-1\right)}\left(k+1\right)-G_{rj}\left(k+1\right){\bar{z}}_{j}\left(k+1\right),$$

(100) $$H_{r}^{\left(j\right)}\left(k+1\right)=H_{r}^{\left(j-1\right)}\left(k+1\right)-G_{rj}\left(k+1\right){\bar{H}}_{j}\left(k+1\right).$$

The covariance of the residual error after the *j*th orthogonalization step is

(101) $$\begin{array}{cc} R_{r}^{\left(j\right)}\left(k+1\right) & =R_{r}^{\left(j-1\right)}\left(k+1\right)\\ \; & \;-F_{rj}\left(k+1\right){\bar{R}}_{j}^{-1}\left(k+1\right)F_{rj}^{T}\left(k+1\right). \end{array}$$

After all preceding predictive-error components have been removed, define

(102) $$\begin{array}{cc} {\bar{z}}_{r}\left(k+1\right) & =z_{r}^{\left(r-1\right)}\left(k+1\right),\\ {\bar{H}}_{r}\left(k+1\right) & =H_{r}^{\left(r-1\right)}\left(k+1\right),\\ {\bar{v}}_{r}\left(k+1\right) & =v_{r}^{\left(r-1\right)}\left(k+1\right),\\ {\bar{R}}_{r}\left(k+1\right) & =R_{r}^{\left(r-1\right)}\left(k+1\right). \end{array}$$

From Equations (96) and (97),

(103) $$\begin{array}{cc} E\left\{v_{r}^{\left(j\right)}\left(k+1\right){\bar{v}}_{j}^{T}\left(k+1\right)\right\} & =F_{rj}\left(k+1\right)\\ \; & \;-G_{rj}\left(k+1\right){\bar{R}}_{j}\left(k+1\right)\\ \; & =0. \end{array}$$

Consequently, after the sequential orthogonalization,

(104) $$\begin{array}{c} E\left\{{\bar{v}}_{r}\left(k+1\right){\bar{v}}_{s}^{T}\left(k+1\right)\right\}=0,\;r\neq s. \end{array}$$

Equation (104) establishes zero cross-covariance rather than statistical independence.

### 4.10. Orthogonalized Joint Representation up to the *n*th Order

Define the stacked orthogonalized predictive-information vector as

(105) $$\begin{array}{c} z_{\mathrm{orth}}^{\left(n\right)}\left(k+1\right)=\mathrm{col}\left\{{\bar{z}}_{1}\left(k+1\right),{\bar{z}}_{2}\left(k+1\right),\ldots ,{\bar{z}}_{n}\left(k+1\right)\right\}. \end{array}$$

The corresponding stacked observation matrix is

(106) $$\begin{array}{c} H_{\mathrm{orth}}^{\left(n\right)}\left(k+1\right)=\mathrm{col}\left\{{\bar{H}}_{1}\left(k+1\right),{\bar{H}}_{2}\left(k+1\right),\ldots ,{\bar{H}}_{n}\left(k+1\right)\right\}. \end{array}$$

The stacked equivalent error is

(107) $$\begin{array}{c} v_{\mathrm{orth}}^{\left(n\right)}\left(k+1\right)=\mathrm{col}\left\{{\bar{v}}_{1}\left(k+1\right),{\bar{v}}_{2}\left(k+1\right),\ldots ,{\bar{v}}_{n}\left(k+1\right)\right\}. \end{array}$$

The orthogonalized joint predictive model is therefore

(108) $$\begin{array}{c} z_{\mathrm{orth}}^{\left(n\right)}\left(k+1\right)=H_{\mathrm{orth}}^{\left(n\right)}\left(k+1\right)x\left(k+1\right)+v_{\mathrm{orth}}^{\left(n\right)}\left(k+1\right). \end{array}$$

According to Equation (104), the covariance of the orthogonalized joint error is

(109) $$\begin{array}{cc} R_{\mathrm{orth}}^{\left(n\right)}\left(k+1\right) & =E\left\{v_{\mathrm{orth}}^{\left(n\right)}\left(k+1\right)v_{\mathrm{orth}}^{(n)T}\left(k+1\right)\right\}\\ \; & =\mathrm{blkdiag}\left[{\bar{R}}_{1}\left(k+1\right),{\bar{R}}_{2}\left(k+1\right),\ldots ,{\bar{R}}_{n}\left(k+1\right)\right]. \end{array}$$

### 4.11. Orthogonalized Polynomial Prediction Fusion up to the nth Order

Applying weighted least squares to Equation (108), the fused state-prediction estimate is

(110) $$\begin{array}{cc} {\hat{x}}^{\left(n\right)}\left(k+1|k\right) & ={\left[H_{\mathrm{orth}}^{(n)T}\left(k+1\right)R_{\mathrm{orth}}^{(n)-1}\left(k+1\right)H_{\mathrm{orth}}^{\left(n\right)}\left(k+1\right)\right]}^{-1}\\ \; & \;\times H_{\mathrm{orth}}^{(n)T}\left(k+1\right)R_{\mathrm{orth}}^{(n)-1}\left(k+1\right)z_{\mathrm{orth}}^{\left(n\right)}\left(k+1\right). \end{array}$$

The corresponding fused prediction-error covariance is

(111) $$\begin{array}{c} P^{\left(n\right)}\left(k+1|k\right)={\left[H_{\mathrm{orth}}^{(n)T}\left(k+1\right)R_{\mathrm{orth}}^{(n)-1}\left(k+1\right)H_{\mathrm{orth}}^{\left(n\right)}\left(k+1\right)\right]}^{-1}. \end{array}$$

Since the orthogonalized covariance matrix is block diagonal, Equation (111) can equivalently be written in additive information form as

(112) $$\begin{array}{cc} {\left[P^{\left(n\right)}\left(k+1|k\right)\right]}^{-1} & =P^{-1}\left(k+1|k\right)\\ \; & \;+\sum_{r=2}^{n}{\bar{H}}_{r}^{T}\left(k+1\right){\bar{R}}_{r}^{-1}\left(k+1\right){\bar{H}}_{r}\left(k+1\right). \end{array}$$

The fused state-prediction estimate can also be expressed as

(113) $$\begin{array}{cc} {\hat{x}}^{\left(n\right)}\left(k+1|k\right) & =P^{\left(n\right)}\left(k+1|k\right)[P^{-1}\left(k+1|k\right)\hat{x}\left(k+1|k\right)\\ \; & \;+\sum_{r=2}^{n}{\bar{H}}_{r}^{T}\left(k+1\right){\bar{R}}_{r}^{-1}\left(k+1\right){\bar{z}}_{r}\left(k+1\right)]. \end{array}$$

### 4.12. Prediction-Enhanced Kalman Filter up to the *n*th Order

The actual measurement equation is

(114) $$\begin{array}{c} y(k+1)=H(k+1)x(k+1)+v(k+1), \end{array}$$

where

(115) $$\begin{array}{c} v\left(k+1\right)∼N\left(0,R(k+1)\right). \end{array}$$

Based on the fused prediction result, the measurement prediction is

(116) $$\begin{array}{c} {\hat{y}}^{\left(n\right)}\left(k+1|k\right)=H\left(k+1\right){\hat{x}}^{\left(n\right)}\left(k+1|k\right). \end{array}$$

The corresponding innovation is

(117) $$\begin{array}{c} {\tilde{y}}^{\left(n\right)}\left(k+1|k\right)=y\left(k+1\right)-{\hat{y}}^{\left(n\right)}\left(k+1|k\right). \end{array}$$

The innovation covariance is

(118) $$\begin{array}{c} S^{\left(n\right)}\left(k+1\right)=H\left(k+1\right)P^{\left(n\right)}\left(k+1|k\right)H^{T}\left(k+1\right)+R\left(k+1\right). \end{array}$$

The corresponding Kalman gain is

(119) $$\begin{array}{c} K^{\left(n\right)}\left(k+1\right)=P^{\left(n\right)}\left(k+1|k\right)H^{T}\left(k+1\right)S^{(n)-1}\left(k+1\right). \end{array}$$

The state estimate is updated as

(120) $$\begin{array}{c} {\hat{x}}^{\left(n\right)}\left(k+1|k+1\right)={\hat{x}}^{\left(n\right)}\left(k+1|k\right)+K^{\left(n\right)}\left(k+1\right){\tilde{y}}^{\left(n\right)}\left(k+1|k\right). \end{array}$$

The corresponding posterior covariance is updated using the Joseph form:

(121) $$\begin{array}{cc} P^{\left(n\right)}\left(k+1|k+1\right) & =\left[I_{d}-K^{\left(n\right)}\left(k+1\right)H\left(k+1\right)\right]P^{\left(n\right)}\left(k+1|k\right)\\ \; & \;\times {\left[I_{d}-K^{\left(n\right)}\left(k+1\right)H\left(k+1\right)\right]}^{T}\\ \; & \;+K^{\left(n\right)}\left(k+1\right)R\left(k+1\right)K^{(n)T}\left(k+1\right). \end{array}$$

As can be seen from the general formulation above, when n=1, the proposed framework reduces to the conventional first-order prediction and Kalman measurement-update structure. When n=2, it reduces to the second-order orthogonalized PEPKF derived above. For n>2, additional higher-order polynomial predictive representations are progressively incorporated into the weighted least-squares fusion, providing a unified extension of the second-order formulation.

**Figure 1:**
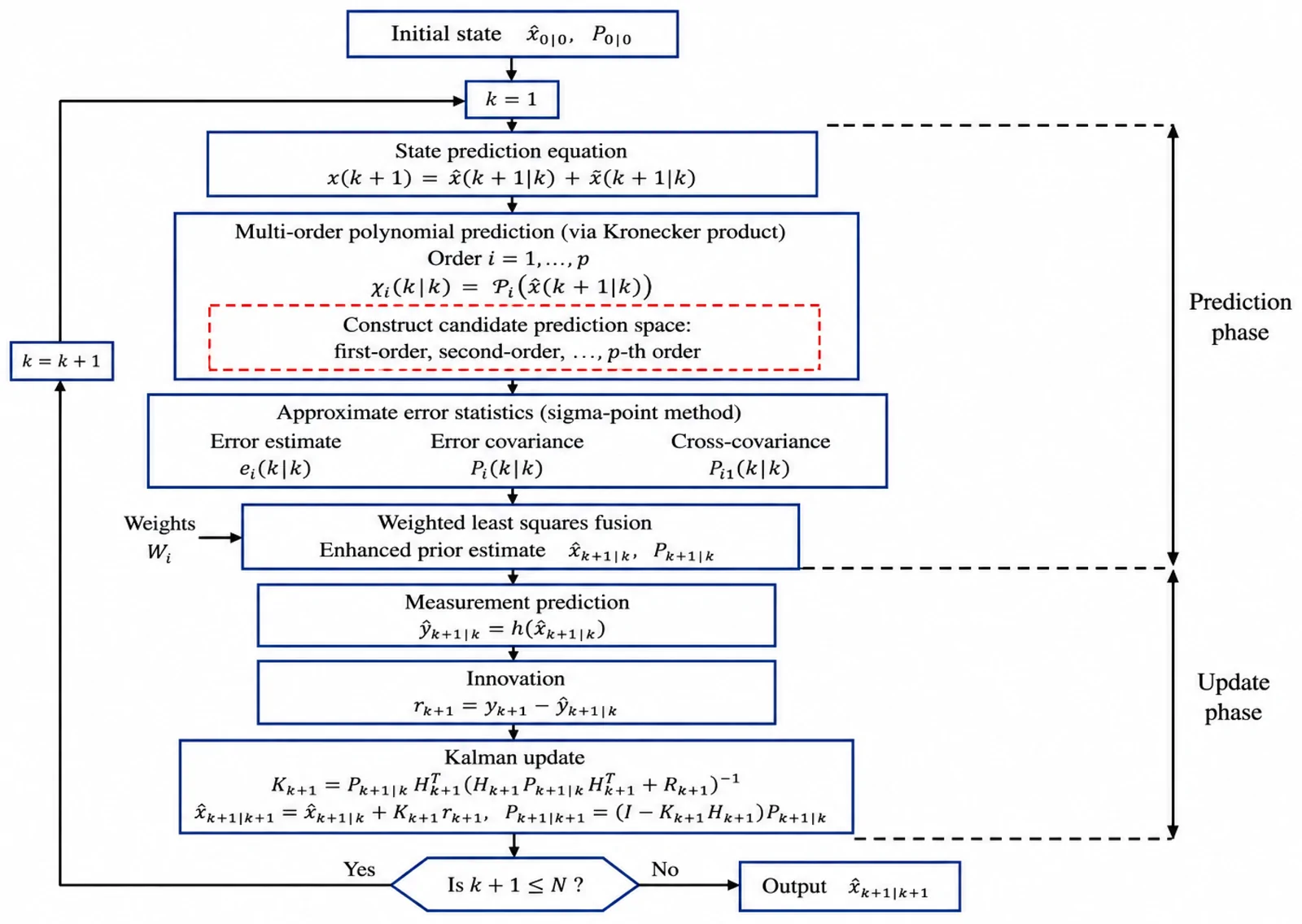
Flowchart of the proposed PEPKF algorithm.

The complete procedure of the proposed method is illustrated in Figure 1.

### 4.13. Computational Complexity and Scalability

For an $n_{x}$-dimensional state, the unrestricted *r*th-order Kronecker representation has dimension $n_{x}^{r}$. Therefore, the corresponding higher-order covariance matrix has dimension $n_{x}^{r}\times n_{x}^{r}$ and requires $O(n_{x}^{2r})$ storage. With $2n_{x}+1$ sigma points, construction of the *r*th-order sigma-point representations scales approximately as $O(n_{x}^{r+1})$. A cross-covariance between the *j*th- and *ℓ*th-order representations has dimension $n_{x}^{j}\times n_{x}^{\ell }$ and requires $O(n_{x}^{j+\ell })$ storage. If a dense $n_{x}^{r}\times n_{x}^{r}$ covariance matrix is inverted directly, the nominal computational complexity is $O(n_{x}^{3r})$.

Consequently, unrestricted Kronecker augmentation becomes computationally expensive for large state dimensions or high polynomial orders. The present study therefore focuses on low-dimensional systems and second- and third-order predictive representations. Although the final fused estimate remains in the original $n_{x}$-dimensional state space, the internal higher-order statistical calculations may dominate the computational cost. Reduced or symmetric Kronecker representations, sparse computation, adaptive order selection, and selective retention of informative higher-order components will be investigated in future work.

The principal dimensional growth and nominal computational costs associated with unrestricted Kronecker augmentation are summarized in Table 1.

As shown in Table 1, the computational burden increases rapidly with both the state dimension $n_{x}$ and the polynomial order *r*. In particular, the dimension of an unrestricted *r*th-order Kronecker representation grows as $n_{x}^{r}$, while the nominal cost of directly inverting the associated dense covariance matrix can scale as $O(n_{x}^{3r})$. Therefore, the present study focuses on second- and third-order predictive representations for low-dimensional systems, for which the computational burden remains manageable.

## 5. Filtering Performance Analysis Under Orthogonalized Predictive Information Fusion

For the joint predictive model composed of the original one-step prediction and the orthogonalized second- through *n*th-order polynomial predictive representations, define the information contribution associated with the *j*th-order orthogonalized pseudo-observation as

(122) $$\begin{array}{c} {\bar{I}}_{j}\left(k+1\right)={\bar{H}}_{j}^{T}\left(k+1\right){\bar{R}}_{j}^{-1}\left(k+1\right){\bar{H}}_{j}\left(k+1\right),\;j=2,\ldots ,n, \end{array}$$

where ${\bar{H}}_{j}\left(k+1\right)$ and ${\bar{R}}_{j}\left(k+1\right)$ denote the equivalent observation matrix and error covariance, respectively, after the sequential Gram–Schmidt orthogonalization.

As derived in the previous section, the equivalent errors associated with the orthogonalized predictive representations satisfy

(123) $$\begin{array}{c} E\left\{{\bar{v}}_{j}\left(k+1\right){\bar{v}}_{\ell }^{T}\left(k+1\right)\right\}=0,\;j\neq \ell . \end{array}$$

Accordingly, the prediction-error covariance obtained from weighted least-squares fusion up to the *n*th order can be expressed as

(124) $$\begin{array}{c} P^{\left(n\right)}\left(k+1|k\right)={\left[P^{-1}\left(k+1|k\right)+\sum_{j=2}^{n}{\bar{I}}_{j}\left(k+1\right)\right]}^{-1}. \end{array}$$

Because each orthogonalized covariance matrix ${\bar{R}}_{j}\left(k+1\right)$ is positive definite,

$${\bar{I}}_{j}\left(k+1\right)={\bar{H}}_{j}^{T}\left(k+1\right){\bar{R}}_{j}^{-1}\left(k+1\right){\bar{H}}_{j}\left(k+1\right)⪰0.$$

Therefore, within the orthogonalized weighted least-squares formulation, the introduction of an additional valid predictive representation adds a positive-semidefinite contribution to the information matrix. Hence, the corresponding prediction covariances satisfy

(125) $$\begin{array}{cc} P^{\left(n\right)}\left(k+1|k\right) & ⪯P^{\left(n-1\right)}\left(k+1|k\right)⪯\ldots \\ \; & ⪯P^{\left(2\right)}\left(k+1|k\right)⪯P\left(k+1|k\right). \end{array}$$

Equation (125) shows that, within the adopted orthogonalized predictive-fusion model, the prediction covariance is non-increasing as valid higher-order predictive representations are incorporated.

For a fixed measurement model H(k+1) and measurement-noise covariance R(k+1), the posterior covariance corresponding to a prior covariance $P^{-}$ can be written in information form as

(126) $$\begin{array}{c} P={\left[{\left(P^{-}\right)}^{-1}+H^{T}\left(k+1\right)R^{-1}\left(k+1\right)H\left(k+1\right)\right]}^{-1}. \end{array}$$

Since the Kalman measurement-update covariance mapping is monotone with respect to the prior covariance in the positive-semidefinite ordering, the ordering in Equation (125) further yields

(127) $$\begin{array}{cc} P^{\left(n\right)}\left(k+1|k+1\right) & ⪯P^{\left(n-1\right)}\left(k+1|k+1\right)⪯\ldots \\ \; & ⪯P^{\left(2\right)}\left(k+1|k+1\right)⪯P^{\left(1\right)}\left(k+1|k+1\right), \end{array}$$

where $P^{\left(1\right)}\left(k+1|k+1\right)$ denotes the posterior covariance obtained using the conventional first-order prediction followed by the standard Kalman measurement update.

Equations (125) and (127) characterize the covariance-contraction property of the proposed orthogonalized weighted least-squares formulation. They show that, within the adopted predictive-fusion model, an effective higher-order predictive representation can contribute additional positive-semidefinite information and consequently reduce the corresponding model-based prediction and posterior covariances.

Within this applicability range, the proposed PEPKF can provide a state-estimation benefit when the enlarged predictive representation space contains informative and non-redundant higher-order components. More specifically, if at least one orthogonalized higher-order predictive block satisfies

(128) $$\begin{array}{c} {\bar{I}}_{j}\left(k+1\right)={\bar{H}}_{j}^{T}\left(k+1\right){\bar{R}}_{j}^{-1}\left(k+1\right){\bar{H}}_{j}\left(k+1\right)\neq 0, \end{array}$$

then the corresponding higher-order representation provides a nonzero information contribution relative to the conventional first-order predictive representation.

For a nonzero state direction *a*, if

(129) $$\begin{array}{c} a^{T}{\bar{I}}_{j}\left(k+1\right)a>0, \end{array}$$

the corresponding higher-order predictive representation contains an effective component in that state direction. Under the validity of the adopted pseudo-observation model, this non-redundant predictive contribution leads to a reduction of the model-based prediction covariance in the corresponding direction. The monotonicity of the subsequent Kalman measurement-update covariance mapping then transfers this reduction to the posterior covariance.

In summary, within the orthogonalized predictive-fusion framework, informative and non-redundant higher-order predictive components provide positive-semidefinite contributions to the information matrix when their statistical characteristics are reliably modeled. Consequently, the model-based prediction covariance is non-increasing as valid higher-order representations are incorporated, and this reduction is further propagated to the posterior covariance through the standard Kalman update. Therefore, under these conditions, PEPKF provides a richer predictive representation than the conventional first-order formulation and can reduce estimation uncertainty by fusing effective higher-order predictive components after correlation removal. This analysis provides a theoretical basis for the performance improvements observed in the subsequent simulations.

## 6. Simulation Experiments and Results Analysis

The estimation performance is mainly evaluated using the root mean square error (RMSE), which is a commonly used metric for quantifying the deviation between the estimated states and the corresponding true states. The RMSE is obtained by first calculating the mean of the squared estimation errors and then taking the square root. A smaller RMSE indicates better estimation performance, as it represents a smaller average deviation between the estimated state and the true state. Specifically, the RMSE is defined as

(130) $$\mathrm{RMSE}=\sqrt{\frac{1}{N}\sum_{k=1}^{N}{\left(x\left(k\right)-\hat{x}\left(k\right)\right)}^{2}},$$

where *N* denotes the total number of sampling instants, x(k) represents the true state at time step *k*, and $\hat{x}\left(k\right)$ denotes the corresponding estimated state.

To evaluate the estimation performance of the proposed PEPKF, simulation experiments were conducted in MATLAB for the considered linear discrete-time systems. The numerical experiments are intentionally conducted on linear discrete-time systems because the physical system class considered in this study is linear. The nonlinear characteristic of PEPKF arises from the internal polynomial predictive transformations introduced through Kronecker augmentation rather than from nonlinear state-transition or measurement equations. Therefore, the simulations are designed to isolate the effect of the nonlinear higher-order predictive representation while keeping the underlying physical state and measurement equations linear.

For each considered case, 100 independent Monte Carlo runs were performed in MATLAB. Each run covered 100 time steps under the same system and filter parameter settings, while the process-noise and measurement-noise sequences were independently regenerated for each realization. The time-varying RMSE curves reported in this section were obtained from the estimation errors at each time step over the 100 independent Monte Carlo runs. For all numerical experiments, the scaled sigma-point parameters are set to α=0.1, β=2, and κ=0. The same parameter setting is used for both the one-dimensional and two-dimensional cases and is kept unchanged throughout all Monte Carlo realizations. With this parameter setting, the central sigma-point weights can be negative. These weights are retained directly according to the standard scaled sigma-point rule, without clipping, truncation, or renormalization, since they are used as deterministic quadrature weights for moment approximation rather than as probability masses.

For the *j*-th state component, the Monte Carlo RMSE at time step *k* is defined as

(131) $${\mathrm{RMSE}}_{j}\left(k\right)=\sqrt{\frac{1}{N_{\mathrm{MC}}}\sum_{r=1}^{N_{\mathrm{MC}}}{\left[x_{j}^{\left(r\right)}\left(k\right)-{\hat{x}}_{j}^{\left(r\right)}\left(k\right)\right]}^{2}},$$

where $N_{\mathrm{MC}}=100$ denotes the number of independent Monte Carlo runs, $x_{j}^{\left(r\right)}\left(k\right)$ is the true value of the *j*-th state component at time step *k* in the *r*-th Monte Carlo realization, and ${\hat{x}}_{j}^{\left(r\right)}\left(k\right)$ is the corresponding state estimate.

### 6.1. Experiment 1

Consider the following one-dimensional discrete-time linear system:

(132) $$\begin{array}{c} \left\{\begin{array}{cc} x(k+1) & =0.1x(k)+w(k),\\ y(k+1) & =0.6x(k+1)+v(k+1). \end{array}\right. \end{array}$$

Here, x(k)∈R denotes the system state at time *k*, and y(k)∈R denotes the system measurement. The state-transition coefficient and measurement coefficient are A=0.1 and H=0.6, respectively. The process noise w(k) and measurement noise v(k) are assumed to be mutually independent zero-mean Gaussian white noises with w(k)∼N(0,Q), v(k)∼N(0,R), where Q=0.5 and R=0.01. The initial state and the initial estimation-error covariance are set to x(0)=2 and P(0|0)=0.001, respectively.

**Figure 2:**
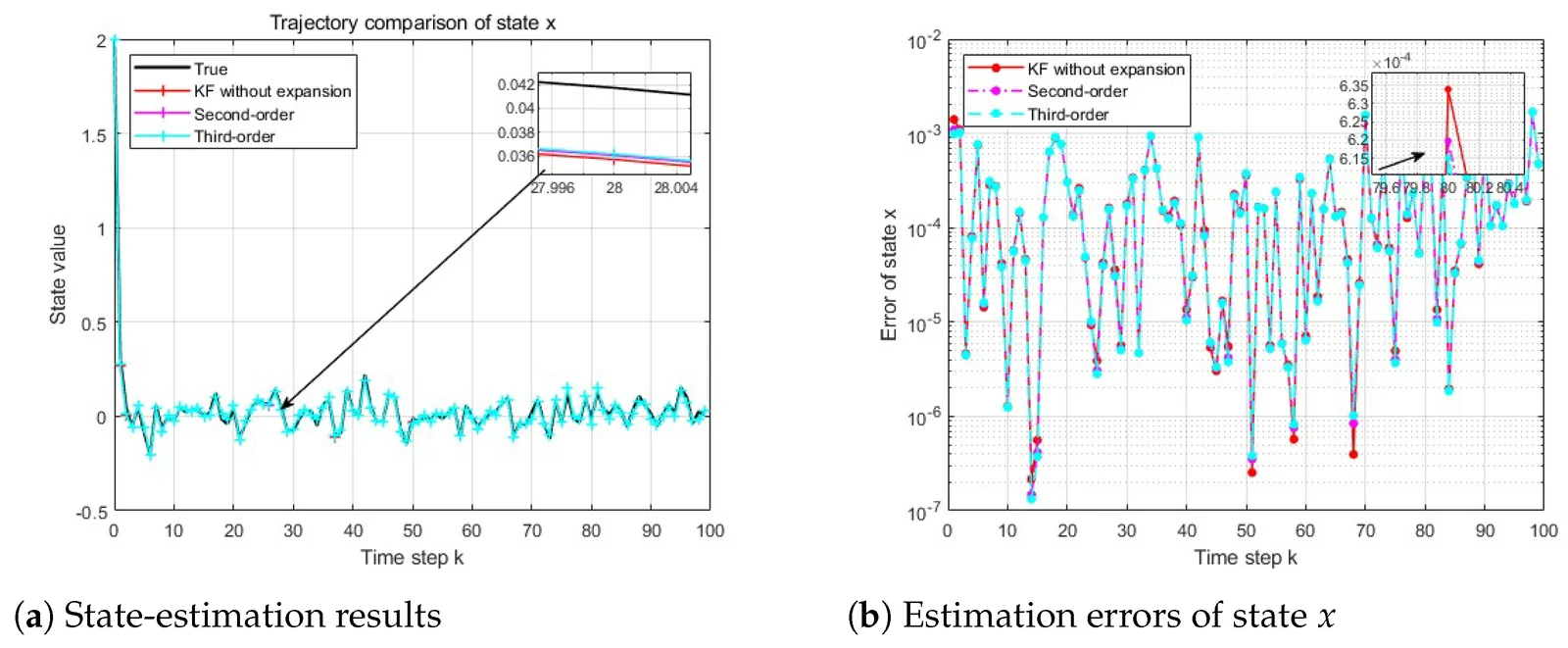
State-estimation results and estimation errors for the one-dimensional system.

**Figure 3:**
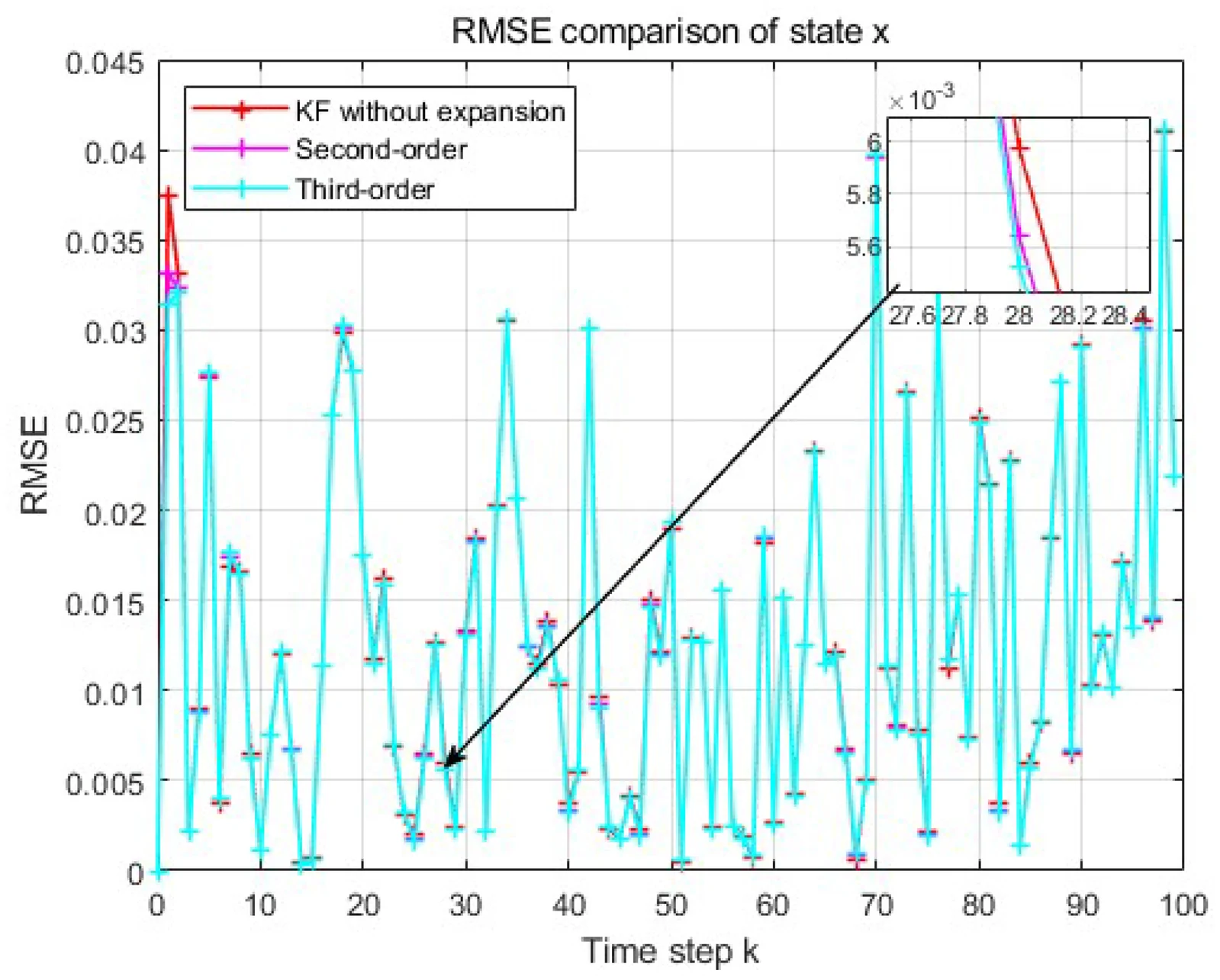
RMSE for State x in a 1D System.

To investigate the effect of the polynomial augmentation order on estimation performance, the conventional KF, the second-order PEPKF, and the third-order PEPKF are applied to the above one-dimensional linear system under identical true-state and measurement sequences. Figure 2 presents the estimation results and errors over 100 time steps, with Figure 2a comparing the true state and the estimated trajectories and Figure 2b showing the corresponding error evolution. Figure 3 further presents the time-varying Root Mean Square Error (RMSE) curves obtained from 100 independent Monte Carlo runs, while Table 2 summarizes the RMSE values and relative improvement rates.

As shown in Figure 2 and Figure 3 and Table 2, the second- and third-order PEPKFs reduce the RMSE of state *x* from 0.01624 for the conventional KF to 0.01610 and 0.01606, corresponding to improvement rates of 0.8259% and 1.104%, respectively. The third-order PEPKF achieves the lowest RMSE, demonstrating the effectiveness of enlarging the first-order prediction space via higher-order Kronecker representations, which yields additional estimation benefits when the components are informative and non-redundant.

### 6.2. Experiment 2

Consider the following two-dimensional discrete-time linear system:

(133) $$\begin{array}{c} \left\{\begin{array}{cc} x(k+1) & =\left[\begin{array}{cc} 0.2 & 0.5\\ 0 & 1 \end{array}\right]x\left(k\right)+w\left(k\right),\\ y(k+1) & =\left[\begin{array}{cc} 1 & 1 \end{array}\right]x\left(k+1\right)+v\left(k+1\right). \end{array}\right. \end{array}$$

The state vector is $x\left(k\right)=\left[\begin{array}{c} x_{1}\left(k\right)\\ x_{2}\left(k\right) \end{array}\right]\in R^{2}$, and y(k)∈R denotes the system measurement. The state-transition and measurement matrices are given by

$$A=\left[\begin{array}{cc} 0.2 & 0.5\\ 0 & 1 \end{array}\right],\;H=\left[\begin{array}{cc} 1 & 1 \end{array}\right].$$

The process noise w(k) and measurement noise v(k) are assumed to be mutually independent zero-mean Gaussian white noises satisfying w(k)∼N(0,Q) and v(k)∼N(0,R), where Q = 0.5 $\left[\begin{array}{cc} 1/3 & 1/2\\ 1/2 & 1 \end{array}\right]$, R=1, and the initial state and initial estimation-error covariance are $x\left(0\right)=\left[\begin{array}{c} 2\\ 2 \end{array}\right]$, $P\left(0|0\right)=0.01I_{2}$.

To investigate the effect of polynomial augmentation order on state-estimation performance, the conventional KF, the second-order PEPKF, and the third-order PEPKF are applied to the above two-dimensional linear system under identical true-state and measurement sequences.

**Figure 4:**
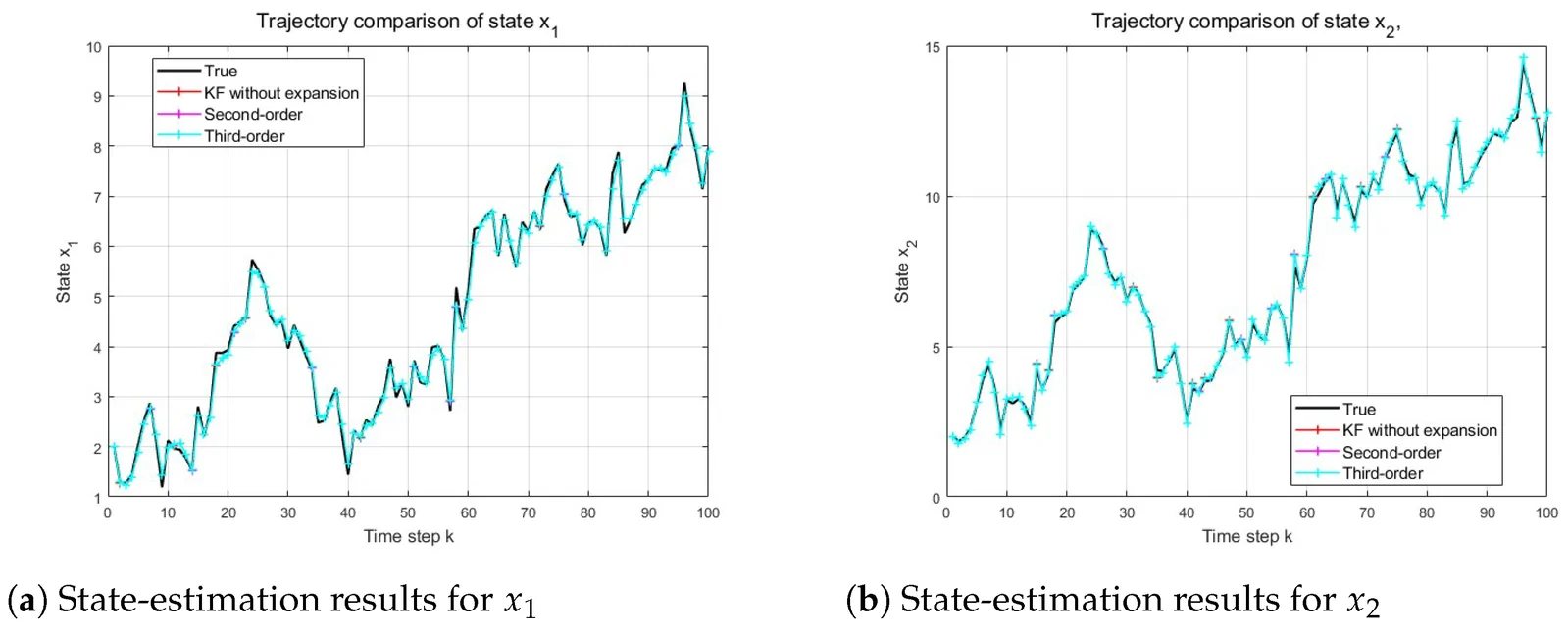
State-estimation results for the two-dimensional system: (**a**) state $x_{1}$; (**b**) state $x_{2}$.

**Figure 5:**
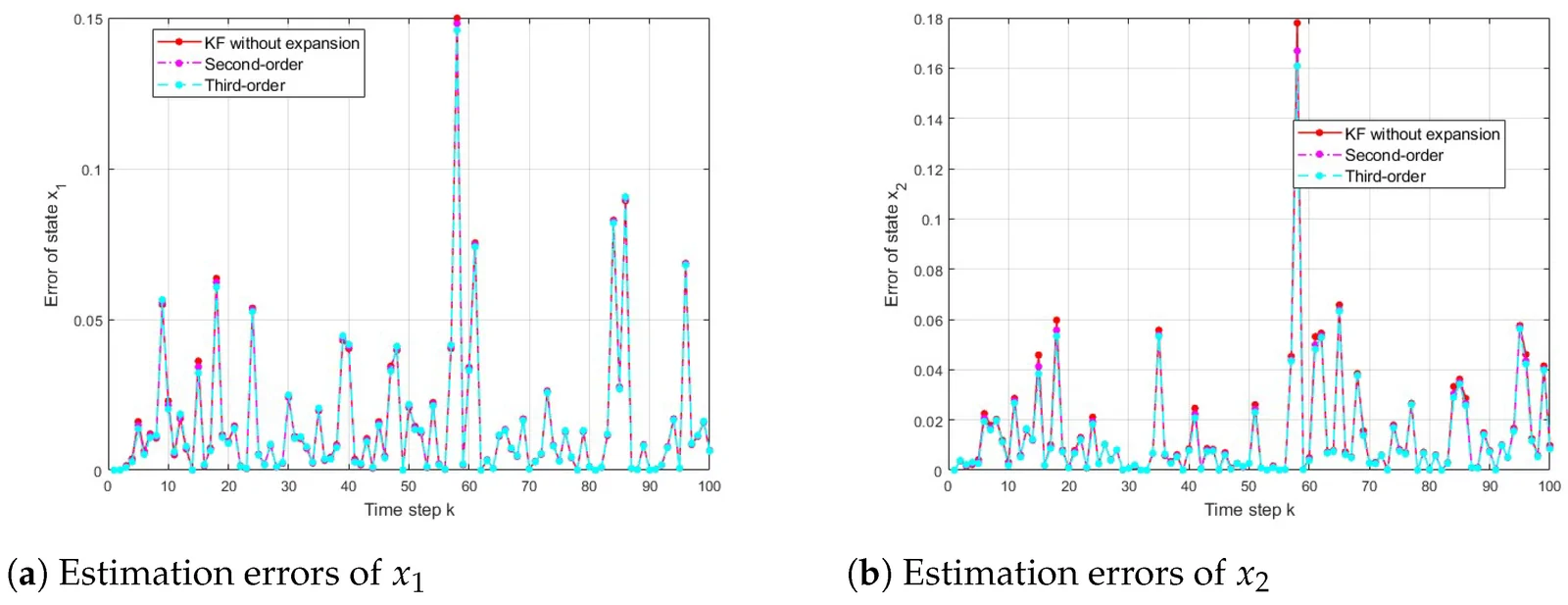
State-estimation errors for the two-dimensional system: (**a**) state $x_{1}$; (**b**) state $x_{2}$.

**Figure 6:**
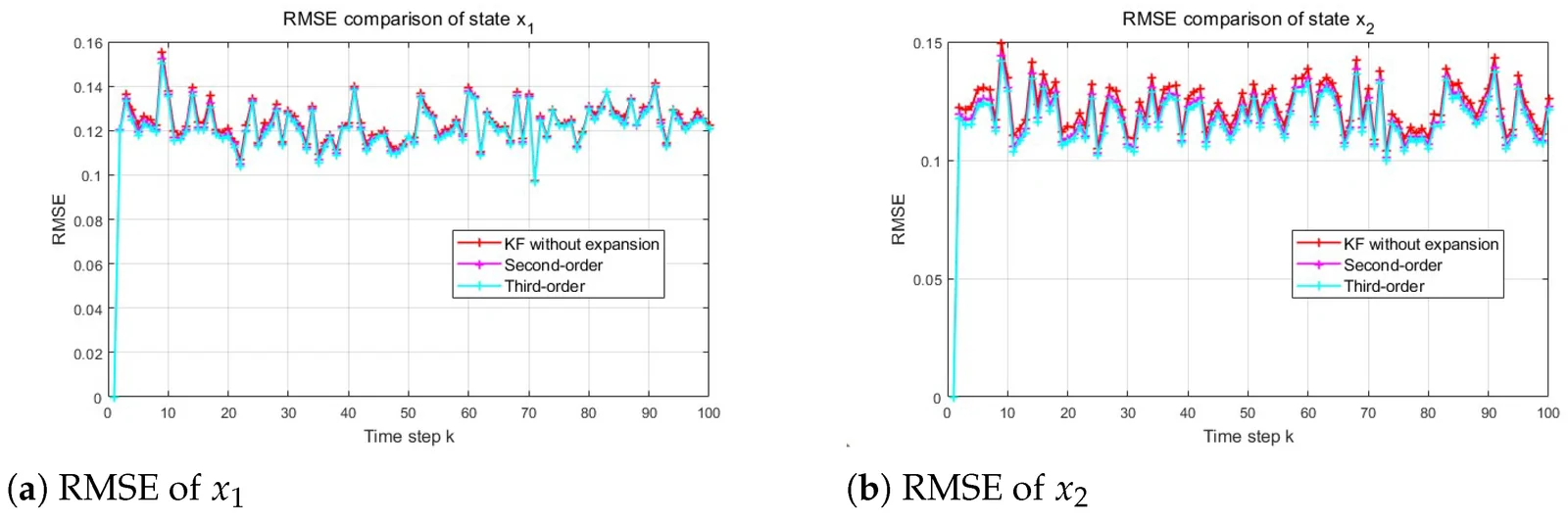
RMSE for States $x_{1}$ and $x_{2}$ in a 2D System: (**a**) state $x_{1}$; (**b**) state $x_{2}$.

Figure 4 and Figure 5 present the state-estimation results and estimation errors over 100 time steps, respectively. The estimation results compare the true states $x_{1}$ and $x_{2}$ with those obtained by the three filters, while the error curves illustrate the corresponding temporal error evolution. Figure 6 further presents the time-varying Root Mean Square Error (RMSE) curves obtained from 100 independent Monte Carlo runs. Table 3 summarizes the corresponding RMSE values and relative improvement rates.

As shown in Figure 4, Figure 5 and Figure 6 and Table 3, the conventional KF achieves RMSE values of 0.1224 and 0.1240 for states $x_{1}$ and $x_{2}$, respectively. The second-order PEPKF reduces the corresponding RMSE values to 0.1208 and 0.1201, representing improvements of 1.307% and 3.145%, respectively, with an average improvement of 2.226%. The third-order PEPKF further reduces the RMSE values to 0.1199 and 0.1183, corresponding to improvements of 2.042% and 4.597%, respectively, with an average improvement of 3.320%. Among the three filters, the third-order PEPKF achieves the lowest RMSE for both state variables. The time-varying RMSE curves further illustrate the corresponding estimation-error behavior over the simulation interval. Compared with the one-dimensional case, the improvement is more evident for the two-dimensional system, in which the state variables exhibit coupled dynamic relationships. This result indicates that the enlarged Kronecker prediction space can explicitly represent higher-order state-coupling and predictive statistical structures that are not directly expressed in the conventional first-order predictive representation. When these higher-order components are reliably characterized and their correlated or redundant parts are removed through Gram–Schmidt orthogonalization, the remaining predictive components can provide useful contributions to the weighted predictive fusion. Consequently, PEPKF can achieve lower empirical estimation errors while retaining the original linear physical model and the standard Kalman measurement-update structure.

Table 4 reports the average computational runtime of the compared filters for the one- and two-dimensional experiments. Each reported value corresponds to one Monte Carlo realization containing 99 filtering-update steps. Noise generation, true-state and measurement-sequence generation, plotting, and post-processing are excluded from the timing measurement.

As expected, the conventional KF requires the least computational time because it operates only in the original first-order state space. In contrast, PEPKF introduces additional computations associated with Kronecker augmentation, sigma-point-based higher-order statistical evaluation, covariance construction, and predictive fusion. In the two-dimensional case, the average runtime increases from approximately 0.50 ms for the conventional KF to 9.88 ms and 19.64 ms for the second- and third-order PEPKF, respectively. Although the higher-order predictive representations increase the computational burden, the measured runtimes remain within the millisecond range for the low-dimensional systems considered in this study, indicating a practical trade-off between computational cost and the use of richer predictive representations.

## 7. Conclusions

This study proposed a Prediction-Enhanced Polynomial Kalman Filter (PEPKF) based on higher-order predictive representations for state estimation. The proposed method enlarges the conventional first-order prediction space by constructing second-, third-, and higher-order polynomial representations through Kronecker augmentation. The resulting higher-order quantities are formulated as equivalent predictive pseudo-observations, whose statistical properties are characterized using sigma-point sampling and subsequently fused with the original one-step prediction through weighted least squares. The standard Kalman measurement-update structure is retained. Because the original and higher-order predictive representations originate from the same prediction process, their statistical correlations are explicitly taken into account before fusion. Theoretical analysis shows that, when the orthogonalized higher-order representations contain informative and non-redundant predictive components and their statistical characteristics are reliably modeled, their incorporation provides positive-semidefinite information contributions and leads to a progressive reduction in the model-based prediction covariance. This covariance reduction is further propagated through the standard Kalman measurement update. Numerical simulation results for one- and two-dimensional systems show that the second- and third-order PEPKFs achieve lower RMSE values than the conventional KF under the considered simulation conditions, with the third-order method providing the best overall estimation performance among the tested methods. These results demonstrate the effectiveness of enlarging the predictive representation space and exploiting higher-order predictive structures for state estimation.

The proposed framework should be interpreted within its applicability range: its performance benefit depends on the presence of informative higher-order predictive structures, accurate covariance and cross-covariance characterization, and sufficiently small approximation errors. Future work will focus on computationally efficient high-dimensional implementations, adaptive selection of polynomial order and predictive representations, systematic comparisons with representative high-order and nonlinear filtering methods, broader robustness evaluations under different process- and measurement-noise covariance levels, statistical covariance-consistency assessments such as NEES analysis, and experimental validation using real sensor systems.

## Figures and Tables

**Table 1:** Computational complexity of the main higher-order quantities in PEPKF.

Quantity	Dimension	Storage/Computational Cost
*r*th-order Kronecker vector	$n_{x}^{r}$	$O(n_{x}^{r})$
*r*th-order covariance matrix	$n_{x}^{r}\times n_{x}^{r}$	$O(n_{x}^{2r})$ storage
Cross-covariance $P^{\left[j,\ell \right]}$	$n_{x}^{j}\times n_{x}^{\ell }$	$O(n_{x}^{j+\ell })$ storage
*r*th-order sigma-point mapping	$\left(2n_{x}+1\right)\times n_{x}^{r}$	$O(n_{x}^{r+1})$
Dense inversion of *r*th-order covariance	$n_{x}^{r}\times n_{x}^{r}$	$O(n_{x}^{3r})$

**Table 2:** PEPKF improvement rate under a one-dimensional linear system.

Filter	RMSE–*x*	Improvement Rate of PEPKF–*x*
KF without augmentation	0.01624	–
Second-order	0.01610	0.8259%
Third-order	0.01606	1.104%

**Table 3:** PEPKF improvement rate under a two-dimensional linear system.

Filter	RMSE–$x_{1}$	RMSE–$x_{2}$	Improvement Rate of PEPKF–$x_{1}$	Improvement Rate of PEPKF–$x_{2}$
KF without augmentation	0.1224	0.1240	–	–
Second-order	0.1208	0.1201	1.307%	3.145%
Third-order	0.1199	0.1183	2.042%	4.597%

**Table 4:** Average computational runtime of the compared filters.

Filter	1D Runtime (s)	2D Runtime (s)
KF without augmentation	$2.1323\times 10^{-4}$	$4.9532\times 10^{-4}$
Second-order PEPKF	$8.0641\times 10^{-3}$	$9.8762\times 10^{-3}$
Third-order PEPKF	$4.1489\times 10^{-3}$	$1.9637\times 10^{-2}$

## Data Availability

The original contributions presented in this study are included in the article. Further inquiries can be directed to the corresponding author.

## Appendix A. Orthogonalized Weighted Least-Squares Derivation for the Second-Order Predictive Model

In the following second-order orthogonalization derivation, the local approximation residual is assumed to be sufficiently small and is therefore neglected. Under this residual-neglected approximation and the centered higher-order error construction, the second-order pseudo-observation error has zero mean.

The original one-step prediction and the second-order pseudo-observation can be jointly written as

(A1) $$\begin{array}{cc} \hat{x}\left(k+1|k\right) & =x\left(k+1\right)+v_{1}\left(k+1\right),\\ {\hat{x}}^{\left[2,1\right]}\left(k+1\right) & =H_{x}^{\left[2,1\right]}\left(k+1\right)x\left(k+1\right)+v_{2}\left(k+1\right), \end{array}$$

where

(A2) $$v_{1}\left(k+1\right)=-\tilde{x}\left(k+1|k\right),$$

and

(A3) $$v_{2}\left(k+1\right)=v_{x}^{\left[2,1\right]}\left(k+1\right).$$

Therefore, the second-order joint model is

(A4) $$\left[\begin{array}{c} \hat{x}\left(k+1|k\right)\\ {\hat{x}}^{\left[2,1\right]}\left(k+1\right) \end{array}\right]=\left[\begin{array}{c} I\\ H_{x}^{\left[2,1\right]}\left(k+1\right) \end{array}\right]x\left(k+1\right)+\left[\begin{array}{c} v_{1}\left(k+1\right)\\ v_{2}\left(k+1\right) \end{array}\right].$$

The first-order prediction error has zero mean,

(A5) $$E\left\{v_{1}\left(k+1\right)\right\}=0,$$

and covariance

(A6) $$R_{1}\left(k+1\right)=E\{v_{1}\left(k+1\right)v_{1}^{T}\left(k+1\right)\}=P\left(k+1|k\right).$$

The second-order pseudo-observation error is defined as

(A7) $$v_{2}\left(k+1\right)=-B^{\left[2,1\right]}\left(k\right)\tilde{x}\left(k+1|k\right)-B^{\left[2,2\right]}\left(k\right){\tilde{\tilde{x}}}^{\left[2\right]}\left(k+1|k\right).$$

Its mean is

(A8) $$E\left\{v_{2}\left(k+1\right)\right\}=0.$$

The covariance matrix of the second-order pseudo-observation error is

(A9) $$\begin{array}{cc} R_{2}\left(k+1\right) & =E\left\{v_{2}\left(k+1\right)v_{2}^{T}\left(k+1\right)\right\}\\ \; & =B^{\left[2,1\right]}\left(k\right)P\left(k+1|k\right)B^{[2,1]T}\left(k\right)\\ \; & \;+B^{\left[2,2\right]}\left(k\right)P_{\tilde{x}\tilde{x}}^{\left[2,2\right]}\left(k+1|k\right)B^{[2,2]T}\left(k\right). \end{array}$$

where the first-order–second-order centered-error cross terms vanish under the zero-mean Gaussian prediction-error assumption because they consist of third-order central moments.

It should be emphasized that this does not imply that $v_{1}\left(k+1\right)$ and $v_{2}\left(k+1\right)$ are uncorrelated. In fact, $v_{2}\left(k+1\right)$ contains the first-order prediction error $\tilde{x}\left(k+1|k\right)$ explicitly. Therefore, their cross-covariance must be considered before the weighted least-squares fusion.

The cross-covariance between the second-order pseudo-observation error and the original one-step prediction error is defined as

(A10) $$F_{21}\left(k+1\right)=E\left\{v_{2}\left(k+1\right)v_{1}^{T}\left(k+1\right)\right\}.$$

Substituting (A2) and (A7) into (A10) gives

(A11) $$\begin{array}{cc} F_{21}\left(k+1\right) & =B^{\left[2,1\right]}\left(k\right)P\left(k+1|k\right)\\ \; & \;+B^{\left[2,2\right]}\left(k\right)P_{\tilde{x}\tilde{x}}^{\left[2,1\right]}\left(k+1|k\right), \end{array}$$

where

(A12) $$P_{\tilde{x}\tilde{x}}^{\left[2,1\right]}\left(k+1|k\right)=E\left\{{\tilde{\tilde{x}}}^{\left[2\right]}\left(k+1|k\right){\tilde{x}}^{T}\left(k+1|k\right)\right\}.$$

Under the zero-mean Gaussian assumption,

(A13) $$P_{\tilde{x}\tilde{x}}^{\left[2,1\right]}\left(k+1|k\right)=0,$$

and therefore

(A14) $$F_{21}\left(k+1\right)=B^{\left[2,1\right]}\left(k\right)P\left(k+1|k\right).$$

To remove the correlation between the two predictive representations, a Gram–Schmidt orthogonalization is applied. Define the orthogonalization matrix as

(A15) $$G_{21}\left(k+1\right)=F_{21}\left(k+1\right)R_{1}^{-1}\left(k+1\right).$$

Since $R_{1}\left(k+1\right)=P\left(k+1|k\right)$, Equation (A14) yields

(A16) $$G_{21}\left(k+1\right)=B^{\left[2,1\right]}\left(k\right).$$

The orthogonalized second-order pseudo-observation error is constructed as

(A17) $${\bar{v}}_{2}\left(k+1\right)=v_{2}\left(k+1\right)-G_{21}\left(k+1\right)v_{1}\left(k+1\right).$$

By substituting $v_{1}\left(k+1\right)=-\tilde{x}\left(k+1|k\right)$ and $G_{21}\left(k+1\right)=B^{\left[2,1\right]}\left(k\right)$, one obtains

(A18) $$\begin{array}{cc} {\bar{v}}_{2}\left(k+1\right) & =-B^{\left[2,1\right]}\left(k\right)\tilde{x}\left(k+1|k\right)-B^{\left[2,2\right]}\left(k\right){\tilde{\tilde{x}}}^{\left[2\right]}\left(k+1|k\right)\\ \; & \;-B^{\left[2,1\right]}\left(k\right)\left[-\tilde{x}\left(k+1|k\right)\right]\\ \; & =-B^{\left[2,2\right]}\left(k\right){\tilde{\tilde{x}}}^{\left[2\right]}\left(k+1|k\right). \end{array}$$

The orthogonality condition follows directly:

(A19) $$\begin{array}{cc} E\left\{{\bar{v}}_{2}\left(k+1\right)v_{1}^{T}\left(k+1\right)\right\} & =F_{21}\left(k+1\right)-G_{21}\left(k+1\right)R_{1}\left(k+1\right)\\ \; & =0. \end{array}$$

The covariance matrix of the orthogonalized second-order error becomes

(A20) $$\begin{array}{cc} {\bar{R}}_{2}\left(k+1\right) & =E\left\{{\bar{v}}_{2}\left(k+1\right){\bar{v}}_{2}^{T}\left(k+1\right)\right\}\\ \; & =R_{2}\left(k+1\right)-F_{21}\left(k+1\right)R_{1}^{-1}\left(k+1\right)F_{21}^{T}\left(k+1\right). \end{array}$$

Under the zero-mean Gaussian condition, this expression can also be written as

(A21) $${\bar{R}}_{2}\left(k+1\right)=B^{\left[2,2\right]}\left(k\right)P_{\tilde{x}\tilde{x}}^{\left[2,2\right]}\left(k+1|k\right)B^{[2,2]T}\left(k\right).$$

For the unrestricted Kronecker representation, $P_{\tilde{x}\tilde{x}}^{\left[2,2\right]}\left(k+1|k\right)$ is generally positive semidefinite rather than necessarily positive definite. This occurs because the full Kronecker representation contains deterministic repeated components. For example, for i≠j,

(A22) $${\tilde{x}}_{i}\left(k+1|k\right){\tilde{x}}_{j}\left(k+1|k\right)={\tilde{x}}_{j}\left(k+1|k\right){\tilde{x}}_{i}\left(k+1|k\right).$$

Such deterministic repetitions generate zero-variance directions in the unrestricted second-order representation. These directions do not provide effective predictive information and are therefore removed before covariance inversion and weighted least-squares fusion. This operation only eliminates the deterministic null directions and does not otherwise alter the higher-order predictive representation.

Let $r_{2}$ denote the rank of the centered second-order covariance matrix,

(A23) $$r_{2}=\mathrm{rank}\left(P_{\tilde{x}\tilde{x}}^{\left[2,2\right]}\left(k+1|k\right)\right).$$

Let $S_{2}\in R^{r_{2}\times n_{x}^{2}}$ be a full-row-rank matrix whose rows form an orthonormal basis for the nonzero-variance subspace of $P_{\tilde{x}\tilde{x}}^{\left[2,2\right]}\left(k+1|k\right)$.

The retained effective second-order centered error is defined as

(A24) $$q_{2,e}\left(k+1\right)=S_{2}{\tilde{\tilde{x}}}^{\left[2\right]}\left(k+1|k\right).$$

Its covariance matrix is

(A25) $$\begin{array}{cc} P_{e}^{\left[2,2\right]}\left(k+1|k\right) & =E\left\{q_{2,e}\left(k+1\right)q_{2,e}^{T}\left(k+1\right)\right\}\\ \; & =S_{2}P_{\tilde{x}\tilde{x}}^{\left[2,2\right]}\left(k+1|k\right)S_{2}^{T}. \end{array}$$

Since $S_{2}$ retains only the nonzero-variance directions, the covariance matrix of the effective second-order representation is strictly positive definite:

(A26) $$P_{e}^{\left[2,2\right]}\left(k+1|k\right)≻0.$$

For the present second-order construction, $B^{\left[2,2\right]}\left(k\right)=I$. Therefore, Equation (A18) reduces to

(A27) $${\bar{v}}_{2}\left(k+1\right)=-{\tilde{\tilde{x}}}^{\left[2\right]}\left(k+1|k\right).$$

Applying the same effective-subspace transformation gives

(A28) $$\begin{array}{cc} {\bar{v}}_{2,e}\left(k+1\right) & =S_{2}{\bar{v}}_{2}\left(k+1\right)\\ \; & =-S_{2}{\tilde{\tilde{x}}}^{\left[2\right]}\left(k+1|k\right)\\ \; & =-q_{2,e}\left(k+1\right). \end{array}$$

Consequently, the covariance matrix of the retained orthogonalized second-order error satisfies

(A29) $$\begin{array}{cc} {\bar{R}}_{2,e}\left(k+1\right) & =E\left\{{\bar{v}}_{2,e}\left(k+1\right){\bar{v}}_{2,e}^{T}\left(k+1\right)\right\}\\ \; & =P_{e}^{\left[2,2\right]}\left(k+1|k\right)≻0. \end{array}$$

Equation (A29) directly proves that, after the deterministic zero-variance directions and the first-order correlated component have been removed, the retained second-order predictive error possesses a positive-definite covariance matrix.

The same result can also be established from the nonsingular transformation structure. Define

(A30) $$e\left(k+1\right)=\tilde{x}\left(k+1|k\right),$$

and

(A31) $$B_{e}^{\left[2,1\right]}\left(k\right)=S_{2}B^{\left[2,1\right]}\left(k\right).$$

The effective second-order error before orthogonalization is

(A32) $$\begin{array}{cc} v_{2,e}\left(k+1\right) & =S_{2}v_{2}\left(k+1\right)\\ \; & =-B_{e}^{\left[2,1\right]}\left(k\right)e\left(k+1\right)-q_{2,e}\left(k+1\right). \end{array}$$

Under the zero-mean Gaussian prediction-error assumption,

(A33) $$\begin{array}{cc} E\left\{e\left(k+1\right)q_{2,e}^{T}\left(k+1\right)\right\} & =E\left\{e\left(k+1\right){\tilde{\tilde{x}}}^{[2]T}\left(k+1|k\right)\right\}S_{2}^{T}\\ \; & =0. \end{array}$$

Define the effective error vector as

(A34) $$\eta_{e}\left(k+1\right)=\left[\begin{array}{c} e(k+1)\\ q_{2,e}\left(k+1\right) \end{array}\right].$$

Its covariance matrix is

(A35) $$R_{\eta ,e}\left(k+1\right)=\left[\begin{array}{cc} P(k+1|k) & 0\\ 0 & P_{e}^{\left[2,2\right]}\left(k+1|k\right) \end{array}\right].$$

Provided that P(k+1|k)≻0, together with $P_{e}^{\left[2,2\right]}\left(k+1|k\right)≻0$, it follows that

(A36) $$R_{\eta ,e}\left(k+1\right)≻0.$$

The first-order and effective second-order errors can be jointly written as

(A37) $$\left[\begin{array}{c} v_{1}\left(k+1\right)\\ v_{2,e}\left(k+1\right) \end{array}\right]=-L_{e}\left(k+1\right)\eta_{e}\left(k+1\right),$$

where

(A38) $$L_{e}\left(k+1\right)=\left[\begin{array}{cc} I & 0\\ B_{e}^{\left[2,1\right]}\left(k\right) & I \end{array}\right].$$

The matrix $L_{e}\left(k+1\right)$ is block lower triangular with identity diagonal blocks and is therefore nonsingular. Hence, the corresponding joint covariance matrix satisfies

(A39) $$\Sigma_{e}\left(k+1\right)=L_{e}\left(k+1\right)R_{\eta ,e}\left(k+1\right)L_{e}^{T}\left(k+1\right)≻0.$$

The effective cross-covariance is

(A40) $$\begin{array}{cc} F_{21,e}\left(k+1\right) & =E\left\{v_{2,e}\left(k+1\right)v_{1}^{T}\left(k+1\right)\right\}\\ \; & =B_{e}^{\left[2,1\right]}\left(k\right)P\left(k+1|k\right). \end{array}$$

Therefore, the corresponding Gram–Schmidt coefficient is

(A41) $$\begin{array}{cc} G_{21,e}\left(k+1\right) & =F_{21,e}\left(k+1\right)R_{1}^{-1}\left(k+1\right)\\ \; & =B_{e}^{\left[2,1\right]}\left(k\right). \end{array}$$

Define the effective Gram–Schmidt transformation matrix as

(A42) $$T_{e}\left(k+1\right)=\left[\begin{array}{cc} I & 0\\ -G_{21,e}\left(k+1\right) & I \end{array}\right].$$

Since $T_{e}\left(k+1\right)$ is block lower triangular with identity diagonal blocks, it is nonsingular. Therefore, the corresponding congruence transformation preserves positive definiteness:

(A43) $$\begin{array}{cc} \; & T_{e}\left(k+1\right)\Sigma_{e}\left(k+1\right)T_{e}^{T}\left(k+1\right)\\ \; & \;=\left[\begin{array}{cc} R_{1}\left(k+1\right) & 0\\ 0 & {\bar{R}}_{2,e}\left(k+1\right) \end{array}\right]≻0. \end{array}$$

It follows again that

(A44) $${\bar{R}}_{2,e}\left(k+1\right)≻0.$$

Therefore, after removing the deterministic zero-variance directions and the component correlated with the original first-order prediction, the retained second-order predictive representation remains non-degenerate and has a positive-definite error covariance. Consequently, ${\bar{R}}_{2,e}^{-1}\left(k+1\right)$ exists and can be used in the subsequent weighted least-squares fusion.

The same orthogonal transformation must be applied to the second-order pseudo-observation equation. Define

(A45) $${\bar{x}}^{\left[2,1\right]}\left(k+1\right)={\hat{x}}^{\left[2,1\right]}\left(k+1\right)-G_{21}\left(k+1\right)\hat{x}\left(k+1|k\right).$$

The corresponding orthogonalized observation matrix is

(A46) $${\bar{H}}_{x}^{\left[2,1\right]}\left(k+1\right)=H_{x}^{\left[2,1\right]}\left(k+1\right)-G_{21}\left(k+1\right).$$

Therefore, the transformed second-order pseudo-observation model becomes

(A47) $${\bar{x}}^{\left[2,1\right]}\left(k+1\right)={\bar{H}}_{x}^{\left[2,1\right]}\left(k+1\right)x\left(k+1\right)+{\bar{v}}_{2}\left(k+1\right).$$

To make the subsequent covariance inversion consistent with the positive-definite effective representation derived above, the same effective-subspace transformation $S_{2}$ is applied to the orthogonalized pseudo-observation equation. Define

(A48) $${\bar{x}}_{e}^{\left[2,1\right]}\left(k+1\right)=S_{2}{\bar{x}}^{\left[2,1\right]}\left(k+1\right),$$

and

(A49) $${\bar{H}}_{x,e}^{\left[2,1\right]}\left(k+1\right)=S_{2}{\bar{H}}_{x}^{\left[2,1\right]}\left(k+1\right).$$

The retained effective second-order pseudo-observation model is therefore

(A50) $${\bar{x}}_{e}^{\left[2,1\right]}\left(k+1\right)={\bar{H}}_{x,e}^{\left[2,1\right]}\left(k+1\right)x\left(k+1\right)+{\bar{v}}_{2,e}\left(k+1\right).$$

Combining the original one-step prediction with the retained effective second-order pseudo-observation gives

(A51) $$\left[\begin{array}{c} \hat{x}\left(k+1|k\right)\\ {\bar{x}}_{e}^{\left[2,1\right]}\left(k+1\right) \end{array}\right]=\left[\begin{array}{c} I\\ {\bar{H}}_{x,e}^{\left[2,1\right]}\left(k+1\right) \end{array}\right]x\left(k+1\right)+\left[\begin{array}{c} v_{1}\left(k+1\right)\\ {\bar{v}}_{2,e}\left(k+1\right) \end{array}\right].$$

According to the orthogonality relation,

(A52) $$E\left\{{\bar{v}}_{2,e}\left(k+1\right)v_{1}^{T}\left(k+1\right)\right\}=0.$$

Therefore, the covariance matrix of the retained transformed joint error is block diagonal:

(A53) $${\bar{R}}_{\mathrm{orth},e}^{\left(2\right)}\left(k+1\right)=\left[\begin{array}{cc} P(k+1|k) & 0\\ 0 & {\bar{R}}_{2,e}\left(k+1\right) \end{array}\right].$$

Since both diagonal blocks are positive definite,

(A54) $${\bar{R}}_{\mathrm{orth},e}^{\left(2\right)}\left(k+1\right)≻0.$$

For compactness, define

(A55) $$z_{\mathrm{orth},e}^{\left(2\right)}\left(k+1\right)=\left[\begin{array}{c} \hat{x}\left(k+1|k\right)\\ {\bar{x}}_{e}^{\left[2,1\right]}\left(k+1\right) \end{array}\right],$$

and

(A56) $$H_{\mathrm{orth},e}^{\left(2\right)}\left(k+1\right)=\left[\begin{array}{c} I\\ {\bar{H}}_{x,e}^{\left[2,1\right]}\left(k+1\right) \end{array}\right].$$

The orthogonalized weighted least-squares problem is then written as

(A57) $$\begin{array}{cc} {\hat{x}}^{\left(2\right)}\left(k+1|k\right)=\arg\min_{x} & {\left[z_{\mathrm{orth},e}^{\left(2\right)}\left(k+1\right)-H_{\mathrm{orth},e}^{\left(2\right)}\left(k+1\right)x\right]}^{T}\\ \; & \times {\left[{\bar{R}}_{\mathrm{orth},e}^{\left(2\right)}\left(k+1\right)\right]}^{-1}\\ \; & \times \left[z_{\mathrm{orth},e}^{\left(2\right)}\left(k+1\right)-H_{\mathrm{orth},e}^{\left(2\right)}\left(k+1\right)x\right]. \end{array}$$

The corresponding normal equation is

(A58) $$\begin{array}{cc} \; & H_{\mathrm{orth},e}^{(2)T}\left(k+1\right){\left[{\bar{R}}_{\mathrm{orth},e}^{\left(2\right)}\left(k+1\right)\right]}^{-1}H_{\mathrm{orth},e}^{\left(2\right)}\left(k+1\right){\hat{x}}^{\left(2\right)}\left(k+1|k\right)\\ \; & \;=H_{\mathrm{orth},e}^{(2)T}\left(k+1\right){\left[{\bar{R}}_{\mathrm{orth},e}^{\left(2\right)}\left(k+1\right)\right]}^{-1}z_{\mathrm{orth},e}^{\left(2\right)}\left(k+1\right). \end{array}$$

Consequently, the orthogonalized second-order predictive estimate is

(A59) $$\begin{array}{cc} {\hat{x}}^{\left(2\right)}\left(k+1|k\right) & ={\left[P^{-1}\left(k+1|k\right)+{\bar{H}}_{x,e}^{[2,1]T}\left(k+1\right){\bar{R}}_{2,e}^{-1}\left(k+1\right){\bar{H}}_{x,e}^{\left[2,1\right]}\left(k+1\right)\right]}^{-1}\\ \; & \;\times [P^{-1}\left(k+1|k\right)\hat{x}\left(k+1|k\right)\\ \; & \;+{\bar{H}}_{x,e}^{[2,1]T}\left(k+1\right){\bar{R}}_{2,e}^{-1}\left(k+1\right){\bar{x}}_{e}^{\left[2,1\right]}\left(k+1\right)]. \end{array}$$

Define the corresponding estimation error as

(A60) $${\tilde{x}}^{\left(2\right)}\left(k+1|k\right)=x\left(k+1\right)-{\hat{x}}^{\left(2\right)}\left(k+1|k\right).$$

Using the retained orthogonalized joint model, the estimation error can be written as

(A61) $$\begin{array}{cc} {\tilde{x}}^{\left(2\right)}\left(k+1|k\right) & =-P^{\left(2\right)}\left(k+1|k\right)[P^{-1}\left(k+1|k\right)v_{1}\left(k+1\right)\\ \; & \;+{\bar{H}}_{x,e}^{[2,1]T}\left(k+1\right){\bar{R}}_{2,e}^{-1}\left(k+1\right){\bar{v}}_{2,e}\left(k+1\right)]. \end{array}$$

where

(A62) $$\begin{array}{cc} P^{\left(2\right)}\left(k+1|k\right)= & [P^{-1}\left(k+1|k\right)\\ \; & \;+{\bar{H}}_{x,e}^{[2,1]T}\left(k+1\right){\bar{R}}_{2,e}^{-1}\left(k+1\right){\bar{H}}_{x,e}^{\left[2,1\right]}\left(k+1\right){]}^{-1}. \end{array}$$

Because the original first-order error and the retained orthogonalized second-order error satisfy

(A63) $$E\left\{v_{1}\left(k+1\right){\bar{v}}_{2,e}^{T}\left(k+1\right)\right\}=0,$$

the covariance matrix of the second-order predictive estimate is

(A64) $$P^{\left(2\right)}\left(k+1|k\right)={\left[P^{-1}\left(k+1|k\right)+{\bar{H}}_{x,e}^{[2,1]T}\left(k+1\right){\bar{R}}_{2,e}^{-1}\left(k+1\right){\bar{H}}_{x,e}^{\left[2,1\right]}\left(k+1\right)\right]}^{-1}.$$
